# T and B cell Epitope analysis of SARS‐CoV‐2 S protein based on immunoinformatics and experimental research

**DOI:** 10.1111/jcmm.16200

**Published:** 2020-12-15

**Authors:** Ziwei Chen, Pinglang Ruan, Lili Wang, Xinmin Nie, Xuejun Ma, Yurong Tan

**Affiliations:** ^1^ Department of Medical Microbiology Xiangya School of Medicine Central South University Changsha China; ^2^ Department of Clinical Laboratory Third Xiangya Hospital Central South University Changsha China; ^3^ Department of NHC Key Laboratory of Medical Virology and Viral Diseases National Institute for Viral Disease Control and Prevention Chinese Center for Disease Control and Prevention Beijing China

**Keywords:** immunoinformatics, molecular dynamics simulation, SARS‐CoV‐2, T and B cell epitopes, T cell immune response, vaccine design

## Abstract

COVID‐19 caused by SARS‐CoV‐2 is pandemic with a severe morbidity and mortality rate across the world. Despite the race for effective vaccine and drug against further expansion and fatality rate of this novel coronavirus, there is still lack of effective antiviral therapy. To this effect, we deemed it necessary to identify potential B and T cell epitopes from the envelope S protein. This can be used as potential targets to develop anti‐SARS‐CoV‐2 vaccine preparations. In this study, we used immunoinformatics to identify conservative B and T cell epitopes for S proteins of SARS‐CoV‐2, which might play roles in the initiation of SARS‐CoV‐2 infection. We identified the B cell and T cell peptide epitopes of S protein and their antigenicity, as well as the interaction between the peptide epitopes and human leucocyte antigen (HLA). Among the B cell epitopes, ‘EILDITPCSFGGVS’ has the highest score of antigenicity and great immunogenicity. In T cell epitopes, MHC‐I peptide ‘KIADYNYKL’ and MHC‐II peptide ‘LEILDITPC’ were identified as high antigens. Besides, docking analysis showed that the predicted peptide ‘KIADYNYKL’ was closely bound to the HLA‐A*0201. The results of molecular dynamics simulation through GROMACS software showed that ‘HLA‐A*0201~peptide’ complex was very stable. And the peptide we selected could induce the T cell response similar to that of SARS‐CoV‐2 infection. Moreover, the predicted peptides were highly conserved in different isolates from different countries. The antigenic epitopes presumed in this study were effective new vaccine targets to prevent SARS‐CoV‐2 infection.

## 1 INTRODUCTION

SARS‐CoV‐2 was discovered in Wuhan City, Hubei Province in early December 2019. This novel coronavirus unlike the previous ones, not only has higher infectivity and pathogenicity, but faster transmission.^1^, ^2^ The present science stood at droplets at the main mode of transmission. Direct contact^3^, ^4^, ^5^, ^6^ as well is common, and some studies have shown that it can be transmitted through faecal‐oral route.^7^ SARS‐CoV‐2 has also been found in urine, but whether the virus can be transmitted through urine remains to be proven.^8^ On 31 January 2020, the World Health Organization declared the COVID‐19 epidemic as a ‘public health emergency of international concern’. This was followed by raising concerns as the global risk level of the epidemic to ‘very high’ on February 28 and defined it as a ‘pandemic’ on March 12. A certain number of asymptomatic and mild patients make prevention and containment more difficult.

SARS‐CoV‐2, a single‐stranded plus‐strand RNA virus, belongs to the genus beta CoV.^9^, ^10^ The four major structural genes encode the nucleocapsid protein (N), the spike protein (S), the envelope protein (E) and the membrane glycoprotein (M); an additional membrane glycoprotein (HE) is present in the HCoV‐OC43 and HKU1 beta‐coronavirus genus.^11^ It has been reported that coronavirus S protein is the main determinant of virus entry into host cells.^12^ Therefore, S protein is an effective choice for vaccine design.

Several platforms are developing SARS‐CoV‐2 vaccine by producing spike subunit, DNA, RNA, whole‐virion or nanoparticle vaccines.^13^ Number of clinical trials and animal model are underway across the world to meet the demand of permanent solution. However, only but few are close clinical significance. Due to the lack of specific drugs and therapies, and the strong communicability of this infection, development of effective vaccines has attracted urgent attention among the scientific community.

Immunoinformatics can be used to predict the conformational (discontinuous) and linear epitopes of viral antigens and to evaluate the immunogenicity and virulence of pathogens. The reverse vaccinology approach has been successful in development of FDA‐licensed vaccines.^14^ Immunoinformatics tools have been used to design a vaccine for MERS‐COV.^15^ The infusion of bioinformatics analysis of vaccine candidates in the decision‐making pipeline could potentially help save billions of dollars and guide to researchers to make an informed choice on selection of candidates. In addition, immunoinformatics methods are time‐saving in designing new vaccines. Using this method, we set the main purpose of this study to identify potential B cell and T cell epitopes from the envelope S protein. This can be used as potential targets to develop anti‐SARS‐CoV‐2 vaccine preparations.

## 2 METHODS

### 2.1 Data retrieval and structural analysis

The primary sequence (QHU79173.2) of S protein was searched from the NCBI database.^16^ The 3D structure (PDB ID:6VXX) of S protein was searched by Protein‐Data‐Bank.^17^ The chemical and physical properties of protein sequences including GRAVY (mean hydrophilicity), half‐life, molecular weight, stability index and atomic composition of amino acids were analysed by online tools Protparam^18^ (https://web.expasy.org/Protparam/). The secondary structure was analysed by PSIPRED^19^ (http://bioinf.cs.ucl.ac.uk/psipred/). Using the TMHMM Server v.2.0 (http://www.cbs.dtu.dk/services/TMHMM/) online tool, the transmembrane topology was checked. The presence of disulphide bonds through the online tool DIANNA v1.1^20^ (http://clavius.bc.edu/~clotelab/DiANNA/) was checked. DiANNA is a unified software for Cysteine state and Disulfide Bond partner prediction. Vaxijen v2.0, the prediction of protective antigens and subunit vaccines, was also used for antigenicity examination.^21^

### 2.2 B cell epitope prediction

The free online access server IEDB^22^ (Immune‐Epitope Database And Analysis‐Resource) was used to predict B cell epitopes. Vaxijen 2.0 server was used to study the antigenicity of selected epitopes. Bepipred linear epitope prediction and Parker hydrophilic prediction algorithm, Kolaskar and Tongaonkar antigenicity scale, Emini surface accessibility prediction tool, and Karplus and Schulz predictability tool were used to analyse hydrophilicity, linear epitope separation, surface accessibility and flexibility.^23^ The β‐rotation in polyproteins was predicted using Chou and Fasman β‐rotation prediction algorithm.^24^ As the discontigous epitopes are clearer and have more advantages than linear epitopes,^25^ the prediction of discontiguous epitopes had been carried out separately through the DiscoTope server.^26^ The parameter was set to ≥0.5. The position of the predicted epitope on the 3D structure of S protein was examined by Pymol.^27^

### 2.3 T cell epitope prediction

Epitopes of cytotoxic T lymphocytes (CTL) are playing a key role in vaccine research. Two online tools, Propred‐1^28^ and Propred,^29^ were used to predict the CTL epitopes of MHC class I and MHC‐II target proteins, respectively. For Propred‐1 proteasome and immune proteasome, the threshold was maintained at 5%.

### 2.4 Eminent features profiling of selected T cell epitopes

After determining the antigenic epitopes of MHC‐1 and II class alleles, the digestion, mutation, toxicity, sensitization, moisture and physicochemical properties of each epitope were detected by Vaxijen 2.0 (http://www.ddgpharmfac.net/Vaxijen/Vaxijen/Vaxijen.html), Protein Digest (http://db.Systemsbiology.net:8080/proteomicsToolkit/proteinDigest.html), AllergenFP 1.0^30^ (http://libdb.csu.edu.cn/rwt/PUBMED/http/MSTGPLLRNBRYE5LGMFST65UFPE/AllergenFP) and ToxinPred server (https://webs.iiitd.edu.in/raghava/toxinpred/design.php). Allergen FP 1.0 is a bioinformatics tool for allergenicity prediction. ToxinPred was used to predict non‐toxic / toxic peptide in order to select non‐toxic epitopes for further analysis.

### 2.5 Conservation analysis of selected epitopes

S protein sequences from 8 different countries were selected from the Genbank database.^31^ Multiple sequence alignment was carried out by Jalview 2.11.0 software to analyse the conservatism of the selected epitopes. At the same time, a phylogenetic tree was established by MEGA7.0 to represent the evolutionary relationship of SARS‐CoV‐2 in 8 different countries. The variability and conservatism of all the selected epitopes were analysed by the results of multiple sequence alignment and the IEDB conservatism analysis tool.

### 2.6 Structural modelling and molecular docking

3D structures of all predicted peptides were modelled on the RPBS MOBYL portal through a PEPFOLD server,^32^ and the 3D structures of MHC molecules including HLA‐A*0201 (PDB ID: 6APN), HLA‐A24 (PDB ID: 3WL9), HLA‐A2.1 (PDB ID: 1EEZ), HLA‐B*2705 (PDB ID: 3LV3), HLA‐B7 (PDB ID: 3VCL), HLA‐B*3501 (PDB ID: 1A9B) are downloaded from the protein database for further molecular docking. Peptides models (antigenic determinants) were docked with MHC molecules by Autodock vina tool to analyse their inhibitory potential.^33^ After removing ligands and water molecules by sequence editor (SEQ), followed by Protonate 3D energy Minimize in previous studies, molecular docking was carried out. The energy approximation of the docking structure can be simulated by using the London‐dG scoring function. Then, the protein‐peptide connection was examined by Ligand Interactions tool. The UCSF Chimera tool is used to generate a PDB model of the predicted peptide 3 D structure.^34^

### 2.7 Molecular dynamic simulation

The analysis of structural stability of the HLA‐A*0201~peptide complex is essential to understand its binding affinity. In the present study, we used GROMACS 2019.6 software (downloaded from www.gromacs.org/) on Linux Centos 7 operating system for molecular dynamics simulation. The structure was processed by pdb2gmx and the force field that we used is the all‐atom OPLS. And then, we started defining the unit cell and adding solvent. The solvated, electroneutral system was then assembled. Before we begin dynamics, we must ensure that the system has no steric clashes or inappropriate geometry. The structure was relaxed through a process called energy minimization (EM). To equilibrate the system, we conducted a 100‐ps NVT equilibration (the NVT equilibration, stabilized the temperature of the system) and a 100‐ps NPT equilibration (the NPT ensemble, wherein the number of particles, pressure, and temperature were all constant) for this system. And then, run production MD for data collection. All data were processed by Originlab (downloaded from www.originlab.com).

### 2.8 Isolation of peripheral blood mononuclear cell (PBMC)

Peripheral blood mononuclear cells from healthy donors were isolated from fresh blood samples using Ficoll‐Paque density gradient centrifugation in our BSL‐3 laboratory at the same day of blood collection. The majority of purified PBMCs were used for immune cell phenotyping where was plasma samples were subjected to antibody testing. The rest of the cells were cryopreserved in freezing medium (90% FBS + 10% DMSO) at 5 × 10^6^ cells/mL at −150°C.

### 2.9 T cell proliferation

Peripheral blood mononuclear cells were cultured in the presence of soluble anti‐CD3 antibody, anti‐CD28 antibody and IL‐2 (15 ng/mL) for 3 days to obtained T cells. To measure T cell proliferation, carboxyfluorescein 6 succinimidyl ester (CFSE, Thermo Scientific)‐labelled PBMCs were cultured in 96‐well U‐bottom plates with RPMI 1640 medium containing 10% FBS and 1% streptomycin/penicillin (all from Gibco). Proliferated T cells were identified by CFSE dilution.

### 2.10 Intracellular cytokine staining (ICS)

To measure T cell activation, PBMCs were stimulated with the commercially available cell activation cocktail (BioLegend) containing phorbol 12‐myristate‐13‐acetate (PMA) and 23 ionomycin in the presence of brefeldin A (BFA, 7.5 µg/mL, Sigma‐Aldrich) for 6 hours. For T cell responses, PBMCs were stimulated with 5 µg/mL of peptides or 5 µg/mL of SARS‐CoV‐2 S protein. Cells were incubated at 37°C overnight and brefeldin A (BFA) was added at 2 hours post‐incubation. S protein of SARS‐CoV‐2 stimulation was included as positive control and no‐peptide stimulation was performed as the negative control.

The required antibodies for flow cytometry are CD3‐BV421(BD Biosciences), CD8‐PE‐Cy7(BD Biosciences), INF‐γ‐APC (BD Biosciences), GZMB‐PerCP (BioLegend) and TNF‐Alexa Fluor 700 (BioLegend).

### 2.11 Western blot analysis and Immunoprecipitation (IP)

Primary T cells stimulated by peptide were collected, washed with cold PBS twice and lysed with NP‐40 lysate at 4°C for 20 minutes. The protein extracts were separated by electrophoresis with 12% prefabricated sodium dodecyl sulphate‐polyacrylamide microgel (SDS‐PAGE) and transferred to PVDF membrane. The membrane was incubated with the indicated antibody and detected by chemiluminescence.

Immunoprecipitation: The total cell lysates were first rotated with the labelled peptide at 4°C overnight. The total protein suspension was then incubated with appropriate antibodies (anti‐HLA‐A2: Abcam, ab74674 and ab168405; anti‐Flag: Cell Signaling Technology, 14793) overnight, and then rotated with protein A/G beads overnight at 4°C. Wash with np‐40 lysis buffer for 3 times, mix with 4 × SDS sample buffer and boil for 10 minutes. Western blot analysis of co‐precipitation.

## 3 RESULT

### 3.1 Structural analysis of S protein

The physical and chemical properties of the S protein calculated by Protparam showed that the protein contained 1273 amino acids (aa), with a molecular weight of 141 204.51 Da and had good antigenicity. Theoretical pI is 6.21 and the isoelectric point under 7 shows negatively charged protein. Among the 1273 residues, the total number of negatively charged residues (Asp + Glu) is 110 and the total number of positively charged residues (Arg + Lys) is 103. The instability index (II) is computed to be 33.01, which means that the protein is very stable. The aliphatic index is 84.67, and the grand average of hydropathicity (GRAVY) is −0.078. The estimated half‐life is as follows: 30 hours (mammalian reticulocytes, in vitro); 20 hours (yeast, in vivo); and 10 hours (Escherichia coli, in vivo). The total number of carbon (C), oxygen (O), nitrogen (N), hydrogen (H) and sulphur (S) is named by the formula C_6339_H_9772_N_1654_O_1895_S_54_. The physicochemical properties of the protein were calculated by Protparam (Table S1). In addition, the secondary and 3‐D structures of S protein by PSIPRED and Pymol, including its Beta sheets, Helixes and Loops structures were shown in Figure S1 and S2, and the positive and negative 3D conformations of S protein were shown in Figure S3.

In addition, the position of the disulphide bond (S‐S) was calculated using DiANNA1.1 tool and assigned a score (Table S2). The high specificity of the protein was evaluated by Vaxijen 2.0 by setting the threshold to ≥0.5. The antigenicity analysis of the full‐length protein showed that the S protein was the expected antigen with a antigenicity of 0.4700. Through TMHMM, an online tool for examining the topology of transmembrane proteins, it was found that residues from 1 to 1213 were exposed on the surface, 1214 to 1236 were inside the transmembrane region, and 1237 to 1273 were buried in the core‐S protein region.

### 3.2 Recognition of B cell epitopes

The primary sequence of S protein was scanned by the IEDB server to predict B cell epitopes. From all the predicted epitopes, 10 epitopes exposed to the surface of S protein with high antigenicity scores were selected (Table 1). Vaxijen 2.0 was used to calculate the scores of antigenicity and the TMHMM server was used to check surface availability (1 to 1213). Among these selected epitopes, ‘EILDITPCSFGGVS’ at position 583 and ‘ILPVSMTKTSVDCT’ at position 726 showed the highest antigenicity and scores. A total of 33 B cell epitopes were obtained, and 10 B cell epitopes with the highest scores were selected.

**Table 1:** B cell epitopes on the surface, starting position and antigenicity score predicted by IEDB analysis of resources and BCPRED

	Start position	Epitopes sequences	Score	Antigenicity
1	416	GKIADYNYKLPDDF	0.9	0.9776
2	374	FSTFKCYGVSPTKL	0.9	0.8708
3	726	ILPVSMTKTSVDCT	0.89	1.606
4	647	AGCLIGAEHVNNSY	0.87	0.829
5	895	QIPFAMQMAYRFNG	0.86	0.7789
6	938	LSSTASALGKLQDV	0.85	0.8753
7	640	SNVFQTRAGCLIGA	0.85	0.7968
8	228	DLPIGINITRFQTL	0.84	1.1058
9	583	EILDITPCSFGGVS	0.81	1.6193
10	539	VNFNFNGLTGTGVL	0.8	1.2915

Note

The predicted B cell epitopes are sorted according to the scores obtained by the trained recurrent neural network. The higher the score, the higher the probability of being an epitope. The higher the score of antigenicity, the more likely it is to be used as an antigen.

**Figure 1:**
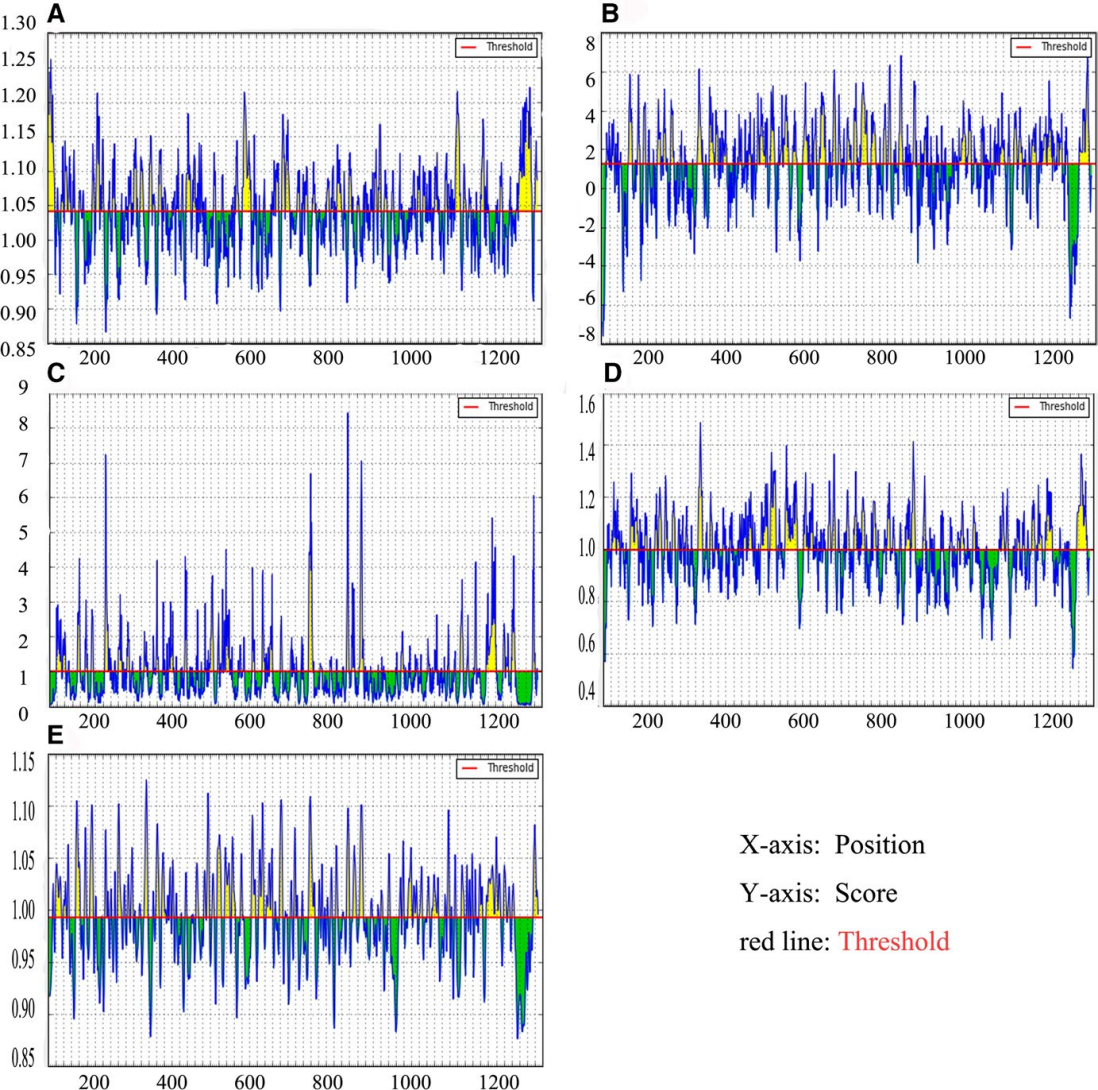
Recognition of B cell epitopes. A, Kolaskar and Tongaonkar antigenicity scales were used to predict antigenic determinants; B, Parker hydrophilicity was used to predict hydrophilicity; C, Emini surface accessibility scale for surface accessibility analysis; D, Chou and Fasman β metamorphosis prediction were used to analyse β variants of structural polyproteins; E, Karplus and Schultz flexibility scale analysis

In order to further check the surface availability of B cell epitopes, Kolaskar and Tongaonkar antigenicity measurement tools were used to analysed B cell epitopes through evaluating the physical and chemical properties of amino acids and their abundance in known B cell epitopes. The higher antigenicity score indicates that it has stronger potential to initiate the immune response. By adjusting the threshold of the tool to 1.041 and keeping the window size to 7, the values of the protein antigen were 1.041 (average), 0.866 (minimum) and 1.261 (maximum) (Figure 1A). In order to find out the surface availability and hydrophilicity of possible B cell epitopes, Parker Hydrophilicity Prediction was used. The values were 1.235 (average), −7.629 (minimum) and 7.743 (maximum). The values of Emini Surface Accessibility Prediction used to analyse surface accessibility (threshold was 1.000) were 1.000 (average), 0.042 (minimum) and 6.047 (maximum) (Figure 1B,C). The results of the Emini surface accessibility analysis can be found in the attached file (Table S3). The values of Chou‐Fasman Beta‐Turn Prediction analysis algorithm used to predict the β‐shift of S protein were 0.997 (average), 0.541 (minimum) and 1.484 (maximum) with the threshold of the tool adjusted to 0.997 (Figure 1D). The results showed that the regions from 250 to 258 amino acids and the regions from 807 to 813 were more likely the B turns in the peptide structure. Karplus and Schulz flexibility analysis tools showed that the amino acid region (PGDSSSG) from 251 to 257 sequences was highly universal (Figure 1E).

**Figure 2:**
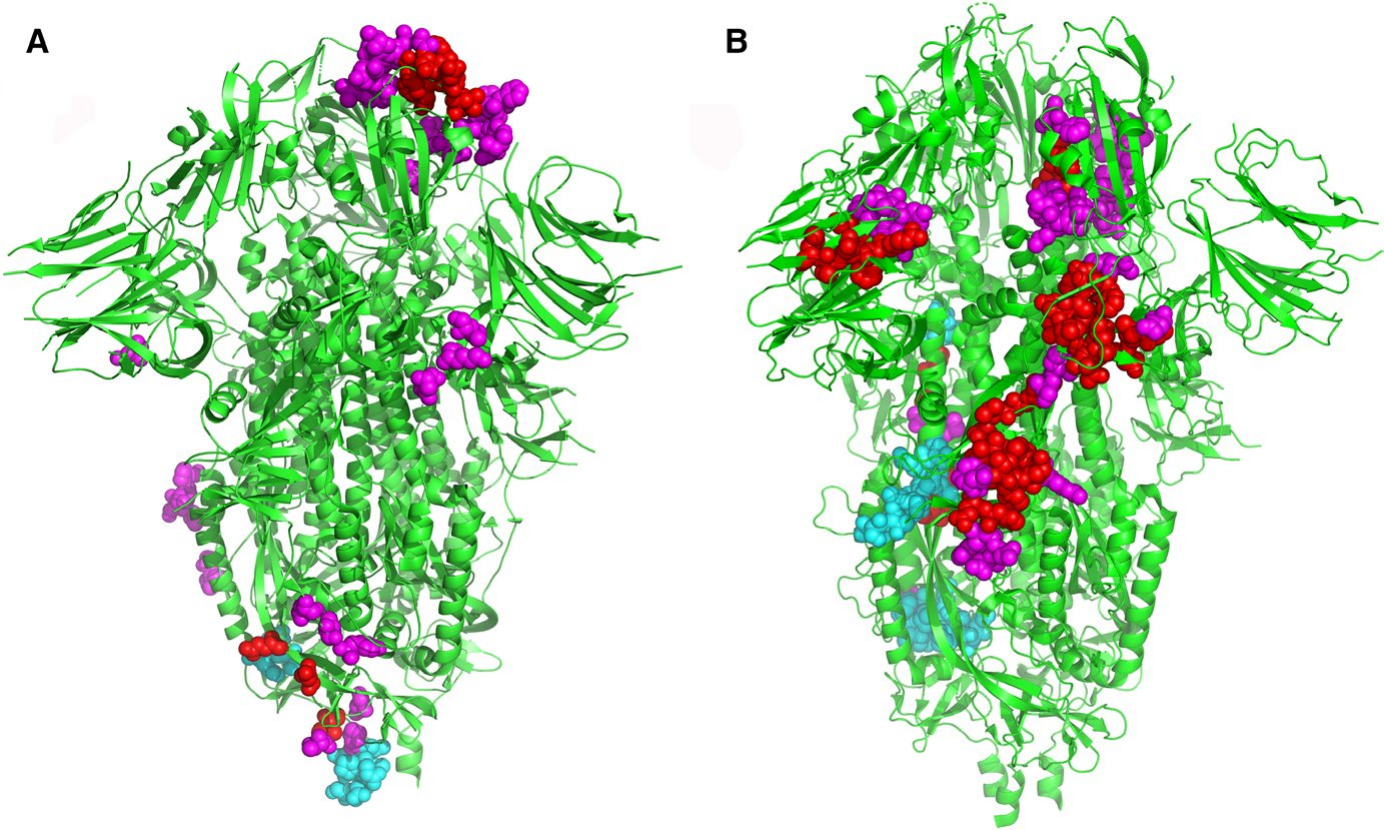
The position of each predicted epitope on the 3D structural surface of S protein was analysed using Pymol. A, the predicted positions of 10 B cell peptide epitopes on S protein were displayed by Pymol software. B, The predicted position of 64 B cell discontinuous epitopes on S protein

In order to further increase the specificity and range of B cell epitopes, the Discotope 2.0 server (http://www.cbs.dtu.dk/services/DiscoTope/) was used to calculate the surface availability according to the number of residue contacts. The 3D structure (PDB ID: 6vxx) of S protein was used to predict discontinuous epitopes with 90% specificity, −3.700 threshold, and 22.000 A tendency score radius. A total of 64 discontiguous epitopes were calculated on different exposed surface areas (Table 2: the first 20 epitopes were shown in Table S4). The position of each predicted epitope on the 3D structural surface of S protein was analysed using Pymol, as shown in Figure 2.

**Table 2:** DiscoTope 2.0 prediction for structure: 6vxx.pdb

Residue ID	Residue name	Contact number	Propensity score	DiscoTope score
282	ASN	5	−2.384	−2.685
415	THR	0	−3.676	−3.253
417	LYS	12	−0.812	−2.099
420	ASP	5	−2.985	−3.217
421	TYR	15	−1.71	−3.238
439	ASN	15	−1.428	−2.989
440	ASN	5	−1.223	−1.657
443	SER	19	0.432	−1.803
444	LYS	8	1.79	0.664
447	GLY	14	2.779	0.849
448	ASN	27	1.336	−1.922
449	TYR	3	0.877	0.431
450	ASN	15	−0.334	−2.021
452	LEU	11	−2.239	−3.247
454	ARG	14	−0.401	−1.965
462	LYS	4	−3.237	−3.324
467	ASP	19	−1.575	−3.579
468	ILE	6	−2.698	−3.078
489	TYR	0	−0.418	−0.37
490	PHE	9	−0.819	−1.76
491	PRO	11	0.123	−1.156

### 3.3 Recognition of T cell epitopes

Propred‐I (47 MHC‐I alleles) and Propred (51 MHC‐II alleles) were used to predict T cell epitopes of S protein. Propred‐I scanned and predicted a library of 47 MHC‐1 class alleles using matrix‐based methods by transferring the S protein sequence of the FASTA format to the Propred‐I server. In addition, antigenicity test and peptide screening were completed with the help of Vaxijen 2.0. According to their antigenicity scores, 10 potential peptides were selected for further analysis (Table 3). Among the predicted epitopes of MHC class I, the peptide ‘KIADYNYKL’ showed a high score of antigenicity (1.6639), with multiple alleles, including HLA‐A2, HLA‐A*0201, HLA‐A*0205, HLA‐A24, HLA‐A3, HLA‐A*3101, HLA‐A2.1, HLA‐B*2705, HLA‐B*3501, HLA‐B*3801, HLA‐B*3902, HLA‐B7 and HLA‐Cw*0401.

**Table 3:** MHC Class I Binding Peptide Prediction Results with their antigenicity scores

	Peptides	MHC class I alleles	Vaxijen score
1	KIADYNYKL	HLA‐A2, HLA‐A*0201, HLA‐A*0205, HLA‐A24, HLA‐A3, HLA‐A*3101, HLA‐A2.1, HLA‐B*2705, HLA‐B*3501, HLA‐B*3801, HLA‐B*3902, HLA‐B7, HLA‐Cw*0401	1.6639
2	TNFTISVTT	HLA‐B*2702	1.3019
3	VVVLSFELL	HLA‐A2, HLA‐A*0205, HLA‐A24, HLA‐A68.1, HLA‐B*3701, HLA‐B*3902、HLA‐B*5301, HLA‐B*51, HLA‐B7, HLA‐Cw*0301, MHC‐Db, MHC‐Db revised, MHC‐Kb, MHC‐Kd	1.0909
4	TLDSKTQSL	HLA‐A2, HLA‐A*0201, HLA‐A*0205, HLA‐A3, HLA‐A2.1, HLA‐B*2705, HLA‐B*3801, HLA‐B*3901, HLA‐B*3902, HLA‐B8, HLA‐Cw*0401, MHC‐Dd	1.0685
5	GKQGNFKNL	HLA‐A2, HLA‐A20 Cattle, HLA‐B*3902, HLA‐Cw*0301、MHC‐Db、MHC‐Db revised、MHC‐Dd、MHC‐Kb	1.0607
6	VRDLPQGFS	HLA‐B*2702, HLA‐B*2705	1.0501
7	PWYIWLGFI	HLA‐A2.1, HLA‐B*5401, HLA‐B*51	1.0343
8	NFGAISSVL	HLA‐A24, HLA‐B*3701, HLA‐B*3801, HLA‐B*3902, HLA‐Cw*0401, HLA‐Cw*0602, MHC‐Kd	0.9894
9	QGFSALEPL	HLA‐B14, HLA‐B*2705, HLA‐B*3901 HLA‐B*3902, HLA‐B40, HLA‐B*5101, HLA‐B*5102, HLA‐B*5103, HLA‐B*5201, HLA‐B*5401, HLA‐B60, HLA‐B7, HLA‐Cw*0401, MHC‐Db revised, MHC‐Dd, MHC‐Kb	0.8462
10	NHTSPDVDL	HLA‐B*3801, HLA‐B*3901, HLA‐B*3902	0.8446

The basic method of the quantitative matrix was used to predict the peptides interacting with MHC‐II alleles. The sequence was provided to Propred with threshold in FASTA format at 4%. The screening was carried out with the help of Vaxijen 2.0, and 10 high‐scoring epitopes were selected (Table 4). Peptide 'YCILEPRSG' was considered to have higher antigenicity because of its high antigenicity score and virtual attachment with a large number of alleles (nearly 22 including DRB1_0102, DRB1_0306, DRB1_0307, DRB1_0308, DRB1_0311, DRB1_0401, DRB1_0404, DRB1_0405, DRB1_0408, DRB1_0410, DRB1_0423, DRB1_0426, DRB1_0813, DRB1_1101, DRB1_1102, DRB1_1104, DRB1_1106, DRB1_1107, DRB1_1128, DRB1_1311, DRB1_1311 and DRB1_1322).

**Table 4:** MHC Class II Binding Peptide Prediction Results with their antigenicity scores

	Peptides	MHC class II alleles	Vaxijen score
1	LEILDITPC	DRB1_0102, DRB1_0306,DRB1_0307,DRB1_0308,DRB1_0311,DRB1_0401,DRB1_0404,DRB1_0405,DRB1_0408,DRB1_0410,DRB1_0423,DRB1_0426,DRB1_0813,DRB1_1101,DRB1_1102,DRB1_1104,DRB1_1106,DRB1_1107,DRB1_1128,DRB1_1311,DRB1_1311,DRB1_1322	1.6390
2	LPVSMTKTS	DRB1_0306,DRB1_0307,DRB1_0308,DRB1_0311,DRB1_0401,DRB1_0402,DRB1_0421,DRB1_0426	1.5550
3	VVFLHVTYV	DRB1_0101,DRB1_0102,DRB1_0102,DRB1_0102,DRB1_0306,DRB1_0307,DRB1_0308,DRB1_0309,DRB1_0311,DRB1_0401,DRB1_0402,DRB1_0404,DRB1_0405,DRB1_0408,DRB1_0410,DRB1_0421,DRB1_0423,DRB1_0426,DRB1_0701,DRB1_0703,DRB1_0802,DRB1_0804,DRB1_0806,DRB1_0813,DRB1_1101,DRB1_1102,DRB1_1104,DRB1_1106,DRB1_1107,DRB1_1114,DRB1_1120,DRB1_1121,DRB1_1128,DRB1_1301,DRB1_1302,DRB1_1304,DRB1_1305,DRB1_1307,DRB1_1311,DRB1_1321,DRB1_1322,DRB1_1323,DRB1_1327,DRB1_1328,DRB1_1501,DRB1_1506	1.5122
4	YYVGYLQPR	DRB1_0309,DRB5_0101,DRB5_0105	1.4692
5	VVLSFELLH	DRB1_0101,DRB1_0102,DRB1_0305,DRB1_0306,DRB1_0307,DRB1_0308,DRB1_0311,DRB1_0401,DRB1_0426,DRB1_0801,DRB1_0817,DRB1_1101,DRB1_1102,DRB1_1104,DRB1_1106,DRB1_1107,DRB1_1114,DRB1_1121,DRB1_1304,DRB1_1307,DRB1_1311,DRB1_1321,DRB1_1322,DRB1_1323,DRB1_1501,DRB1_1502,DRB1_1506	1.4090
6	VVIGIVNNT	DRB1_0101,DRB1_0102,DRB1_0102,DRB1_0102,DRB1_0306,DRB1_0307,DRB1_0308,DRB1_0309,DRB1_0311,DRB1_0401,DRB1_0402,DRB1_0404,DRB1_0405,DRB1_0408,DRB1_0410,DRB1_0421,DRB1_0423,DRB1_0426,DRB1_0804,DRB1_0806,DRB1_0813,DRB1_1102,DRB1_1107,DRB1_1114,DRB1_1120,DRB1_1121,DRB1_1301,DRB1_1302,DRB1_1304,DRB1_1322,DRB1_1323,DRB1_1327,DRB1_1328,DRB1_1501,DRB1_1506	1.3606
7	YVGYLQPRT	DRB1_0801,DRB1_0802,DRB1_0806,DRB1_0813	1.3483
8	VNLTTRTQL	DRB1_0701,DRB1_0703,DRB1_1501,DRB1_1506	1.3468
9	IGINITRFQ	DRB1_0305,DRB1_0306,DRB1_0307,DRB1_0308,DRB1_0311,DRB1_0401,DRB1_0402,DRB1_0402,DRB1_0405,DRB1_0408,DRB1_0410,DRB1_0421,DRB1_0423,DRB1_0426,DRB1_0801,DRB1_0802,DRB1_0806,DRB1_0813,DRB1_1101,DRB1_1102,DRB1_1104,DRB1_1106,,DRB1_1107,DRB1_1114,DRB1_1120,DRB1_1121,DRB1_1301,DRB1_1302,DRB1_1304,DRB1_1307,DRB1_1311,DRB1_1321,DRB1_1322,DRB1_1323,DRB1_1327,DRB1_1328	1.3386
10	LVKNKCVNF	DRB1_0101,DRB1_0102,DRB1_0301,DRB1_0309,DRB1_0421,DRB1_0701,DRB1_0703,DRB1_1120,DRB1_1301,DRB1_1302,DRB1_1327,DRB1_1328	1.3263

### 3.4 Eminent features profiling of selected T cell epitopes

In order to support our findings, we analysed some important features of the selected epitopes. Peptides digested by fewer enzymes are highly stable and more favourable vaccine candidates. The protein digestion server showed a total of 13 digesting enzyme sites. Allergen FP was used to predict the allergenicity of epitopes, which was a novel method to classify proteins into allergens and non‐allergens according to Tanimoto coefficients. ToxinPred was used to predict the toxicity of the selected epitopes. All T cell epitopes along with their digestion, mutation, toxicity, allergenicity, hydro and physiochemical results were given in Table 5.

**Table 5:** Digestion, Mutation, toxicity, allergenicity, hydro and physiochemical profiling of selected peptides

		Peptides	Non‐digesting enzymes	Mutation position	Toxicity	Allergenicity	Hydrophobicity	Hydrophilicity	Charge	PI	Mass
MHC‐I	1	KIADYNYKL	Clostripain,Cyanogen_Bromide,IodosoBenzoate,Proline_Endopept,Staph_Protease,Trypsin_R	NM	NT	A	−0.22	0.06	1	8.76	1127.31
	2	TNFTISVTT	Clostripain,Cyanogen_Bromide,IodosoBenzoate,Proline_Endopept,Staph_Protease,Trypsin_K,Trypsin_R,AspN	NM	NT	NA	0.03	−0.77	0	5.88	983.09
	3	VVVLSFELL	Trypsin,Clostripain,Cyanogen_Bromide,IodosoBenzoate,Proline_Endopept,Trypsin_K,Trypsin_R,AspN	NM	NT	A	0.33	−1.01	−1	4.00	1018.26
	4	TLDSKTQSL	Chymotrypsin,Clostripain,Cyanogen_Bromide,IodosoBenzoate,Proline_Endopept,Staph_Protease,Trypsin_R	NM	NT	A	−0.26	0.27	0	6.19	992.09
	5	GKQGNFKNL	Clostripain,Cyanogen_Bromide,IodosoBenzoate,Proline_Endopept,Staph_Protease,Trypsin_R,AspN ,Elastase	NM	NT	NA	−0.3	0.26	2	10.02	1005.14
	6	VRDLPQGFS	Cyanogen_Bromide,IodosoBenzoate,Staph_Protease,Trypsin_K	NM	NT	NA	−0.18	0.08	0	6.19	1018.14
	7	PWYIWLGFI	Trypsin,Clostripain,Cyanogen_Bromide,Staph_Protease,Trypsin_K,Trypsin_R,AspN	NM	NT	NA	0.38	−1.89	0	5.88	1194.44
	8	NFGAISSVL	Trypsin,Clostripain,Cyanogen_Bromide,IodosoBenzoate,Proline_Endopept,Staph_Protease,Trypsin_K,Trypsin_R,AspN	NM	NT	NA	0.18	−0.81	0	5.88	907.03
	9	QGFSALEPL	Trypsin,Clostripain,Cyanogen_Bromide,IodosoBenzoate,Trypsin_K,Trypsin_R,AspN	NM	NT	NA	0.05	−0.34	−1	4.00	961.08
	10	NHTSPDVDL	Trypsin,Chymotrypsin,Clostripain,Cyanogen_Bromide,IodosoBenzoate,Staph_Protease,Trypsin_K,Trypsin_R,Chymotrypsin(modified)	NM	NT	A	−0.21	0.26	−1.5	4.20	997.03
MHC‐II	1	LEILDITPC	Trypsin,Chymotrypsin,Clostripain,Cyanogen_Bromide,IodosoBenzoate,Trypsin_K,Trypsin_R	NM	NT	A	0.11	−0.29	−2	3.67	1016.22
	2	LPVSMTKTS	Chymotrypsin,IodosoBenzoate,Staph_Protease,Trypsin_R,AspN,Chymotrypsin(modified)	NM	NT	A	−0.08	−0.2	1	9.11	963.16
	3	VVFLHVTYV	Trypsin,Clostripain,Cyanogen_Bromide,IodosoBenzoate,Proline_Endopept,Staph_Protease,Trypsin_K,Trypsin_R,AspN	NM	NT	A	0.3	−1.5	0.5	7.09	1076.30
	4	YYVGYLQPR	Trypsin,Clostripain,Cyanogen_Bromide,IodosoBenzoate,Staph_Protease,Trypsin_K,Trypsin_R,AspN	NM	NT	NA	−0.14	−0.78	1	8.83	1158.32
	5	VVLSFELLH	Trypsin,Clostripain,Cyanogen_Bromide,IodosoBenzoate,Proline_Endopept,Trypsin_K,Trypsin_R,AspN	NM	NT	NA	0.22	−0.9	−0.5	5.25	1056.27
	6	VVIGIVNNT	Trypsin,Chymotrypsin,Clostripain,Cyanogen_Bromide,IodosoBenzoate,Proline_Endopept,Staph_Protease,Trypsin_K,Trypsin_R,AspN,Chymotrypsin(modified)	NM	NT	NA	0.2	−0.9	0	5.88	928.10
	7	YVGYLQPRT	Cyanogen_Bromide,IodosoBenzoate,Staph_Protease,Trypsin_K,AspN	NM	NT	NA	−0.16	−0.57	1	8.93	1096.25
	8	VNLTTRTQL	Chymotrypsin,Cyanogen_Bromide,IodosoBenzoate,Proline_Endopept,Staph_Protease,Trypsin_K,AspN	NM	NT	NA	−0.23	−0.32	1	10.11	1045.20
	9	IGINITRFQ	Cyanogen_Bromide,IodosoBenzoate,Proline_Endopept,Staph_Protease,Trypsin_K,AspN	NM	NT	NA	−0.03	−0.54	1	10.11	1061.25
	10	LVKNKCVNF	Chymotrypsin,Clostripain,Cyanogen_Bromide,IodosoBenzoate,Proline_Endopept,Staph_Protease,Trypsin_R,AspN	NM	NT	A	−0.14	−0.21	2	9.36	1064.31

Abbreviations: NA PROBABLE NO‐ALLERGEN, A PROBABLE ALLERGEN and the non‐digesting enzymes showing those enzymes which do not digest peptides into fragments; NM, No Mutation; NT, Non‐Toxin; T, toxic.

### 3.5 Conservation analyses of selected epitopes

**Figure 3:**
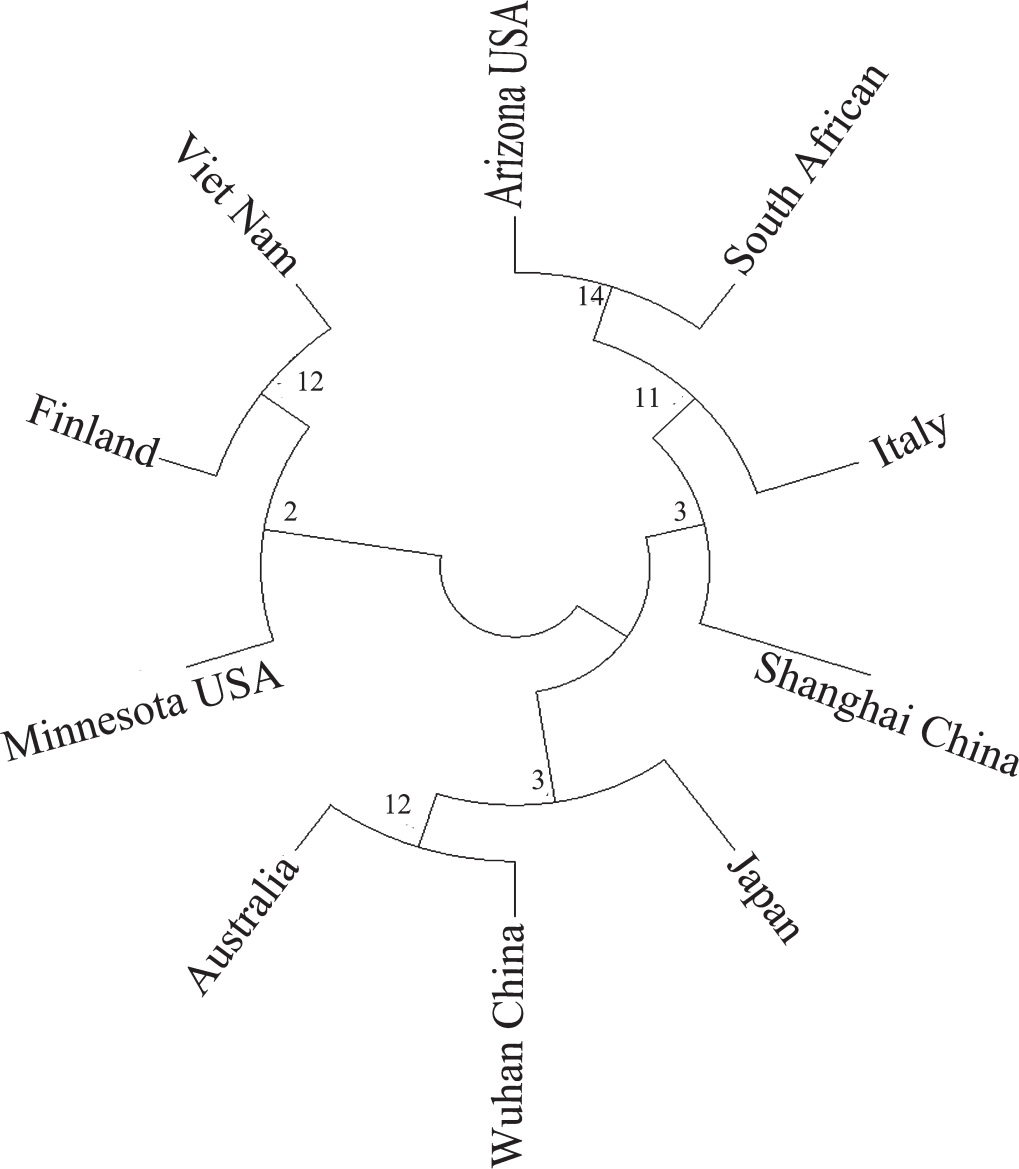
The phylogenetic tree showed that the S protein of SARS‐CoV‐2 isolates from 8 different countries was highly conserved

Isolates of S protein sequences from 8 different countries, including China (Wuhan‐YP_009724390.1, Shanghai‐QII57161.1), Japan (BCB15090.1), Australia (QHR84449.1), United State (Arizona‐QIZ97062.1, Minnesota‐QIK02964.1), Finland (QHU79173.2), Italy (QIA98554.1), Viet Nam (QIK50448.1) and South African (QIZ15537.1) were used for multiple sequence alignment by Jalview 2.11.0 (Figure S4). Analysis the conservatism of the selected epitopes were analysed. As predicted, all selected epitopes were highly conservative as shown in Table S5. At the same time, a phylogenetic tree was established by MEGA7.0 to represent the evolutionary relationship of SARS‐CoV‐2 from 8 different countries (Figure 3). The epitope protection study conducted by the IEDB epitope protection analysis tool showed that, the selected B cell and T cell (MHC‐I class II) epitopes had 100% homology, as shown in Table S5.

### 3.6 Interaction of predicted peptides with HLA alleles

**Figure 4:**
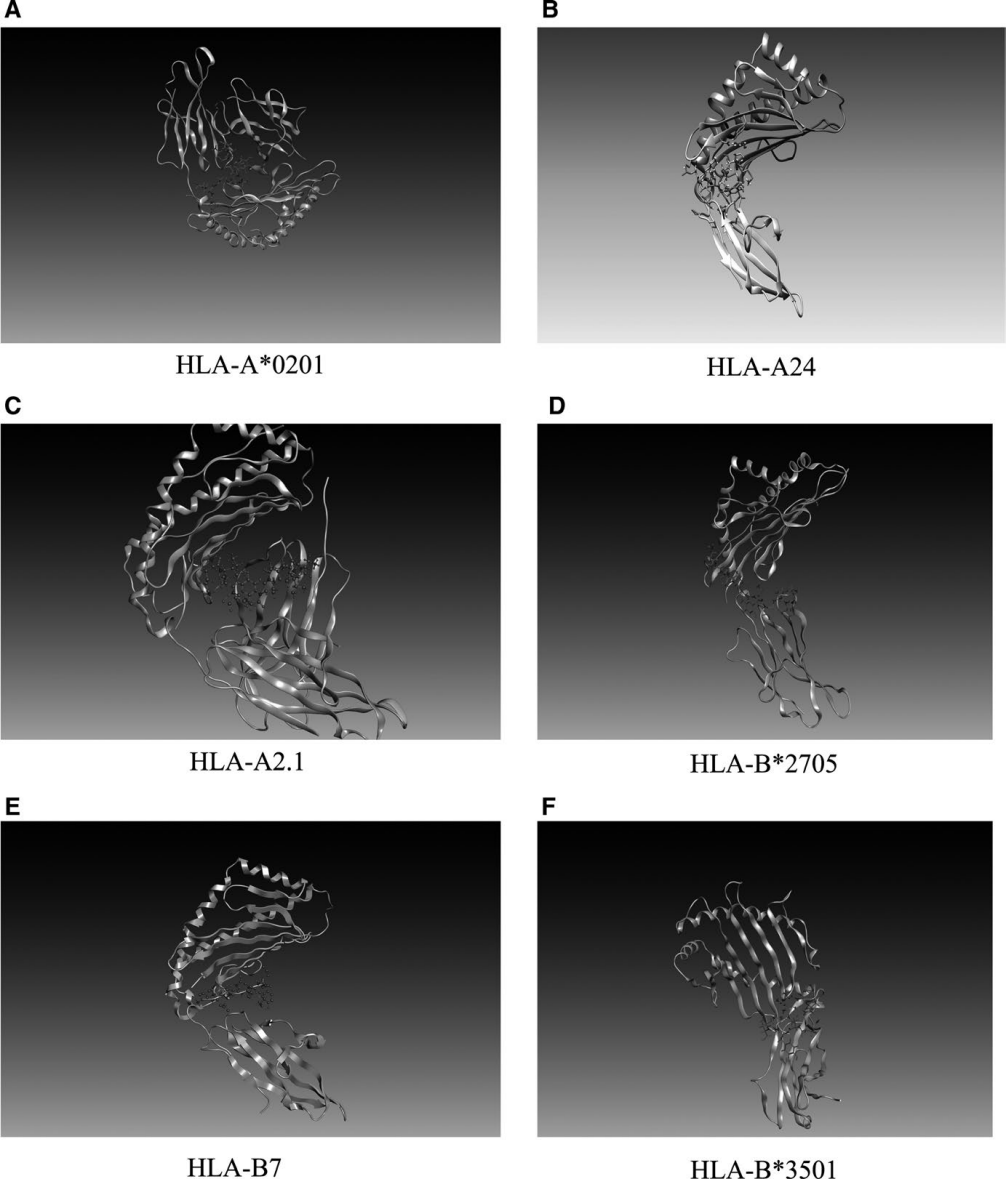
3D graphical representation of the interaction between peptides ‘KIADYNYKL’ and MHC‐I molecules

According to the antigenicity of MHC type I allele and binding peptides predicted by Propred‐I, and in order to further improve the effectiveness of the predicted peptide epitopes, we conducted molecular modelling and molecular docking between the peptide epitopes and their predicted MHC molecules. The 3D structures of two selected MHC‐I binding peptides were predicted by PEPFOLD. It creates five models for each peptide. The best model for each peptide was chosen (Figure S5). According to the affinity between the same peptide and different MHC molecules, we selected MHC class I molecules such as HLA‐A*0201, HLA‐A24 and HLA‐B7, which were docked with the highest antigenic peptide epitope ‘KIADYNYKL’ when compared with other peptide epitopes. Then, the water molecules and ligands were removed by Pymol, leaving the 3D structure of MHC molecules. The model was refined by deprotonation and energy minimization to dock the structure containing peptides with every molecule and the 3D graphical representation of the interaction was shown in Figure 4. Among them, ‘KIADYNYKL’ had the highest docking score with HLA‐A*0201(−10.5433), and the interacting residues were ‘Arg36, Phe46, Asp137, Arg191, Glu320’. At the same time, the docking score of ‘KIADYNYKL’ was also higher than other peptide epitopes. (Table 6).

**Table 6:** Docking results of predicted peptide epitopes and MHC class I molecules

Peptides	MHC class I molecules	Docking score	Interacting residues
KIADYNYKL	HLA‐A*0201	−10.5433	Arg36, Phe46, Asp137, Arg191, Glu320
	HLA‐A24	−10.4514	Gln32, Glu46, Arg48
	HLA‐A2.1	−10.2533	Ser2, Arg6, Tyr27, Asp29, Thr223
	HLA‐B*2705	−10.2093	Glu128, Ala211, Thr214, Thr233
	HLA‐B7	−9.2507	Tyr27, Asp30, Glu212, Thr233, Glu264
	HLA‐B*3501	−8.6753	Thr200, Glu229, Glu232, Arg234, Trp244
TLDSKTQSL	HLA‐A*0201	−9.7071	Glu175, Pro190, Gly380
VVVLSFELL	HLA‐A24	−8.5499	Ser4, Asp29, Asp30, Ser105, Ile213

### 3.7 ‘MHC‐I ‐peptide epitope‐T cell TCR receptor’ complex

**Figure 5:**
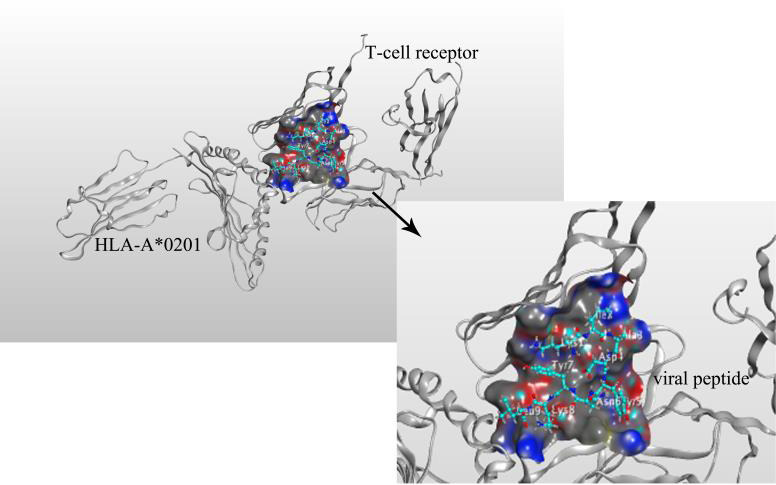
Mock docking of ‘MHC‐I molecule‐peptide epitope‐T cell TCR receptor’ multimer complex

Considering the biological function of MHC, which form complexes with peptide epitopes, and then is presented to T cells, thus initiating specific immune response, we also simulated the ‘MHC‐I ‐peptide epitope‐T cell TCR receptor’ polymer complex. We downloaded the 3D structure (PDB ID:1AO7) of T cell receptors from the PDB database, a complex between human T cell receptors, viral peptide (TAX) and HLA‐A*0201.^35^ Similarly, we did molecular docking through Autodock vina tool. We bind the peptide ‘KIADYNYKL’ to the T cell receptor (Figure 5) with a docking fraction of −9.7562. The interacting residues include the A chain ‘Asp57 and Ile46’ of the T cell receptor and the ‘GLu3, Arg102 and Tyr107’ of the B chain.

### 3.8 Analysis of HLA‐A2 interacting with the peptide

**Figure 6:**
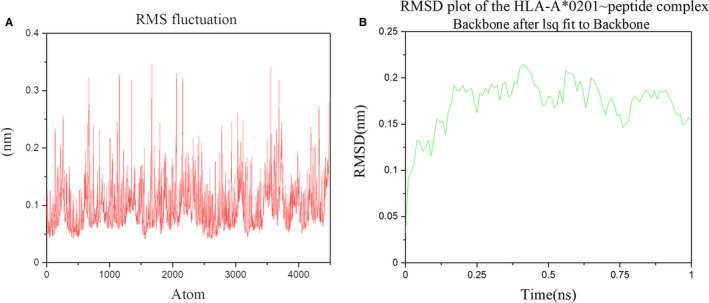
Root mean square fluctuations (RMSF) graph and root mean square deviation (RMSD) graph of the ‘HLA‐A*0201~peptide’ complex

We verified the interaction of HLA‐A2 with the peptide by Molecular dynamic simulation and Immunoprecipitation (IP). RMSF means to take samples for a certain period of time, calculate the average position of each atom during this period of time and then find the square deviation of the atom position during this time. Our result showed that the RMSF was at a very stable value (0.1nm) (Figure 6A).

This result showed that the RMSD level was as low as ~0.175 nm (1Å), indicating that the structure was very stable. The slight difference between the curves showed that the structure at t = 0 ns (RMSD = 0.0327 nm) was slightly different from the crystal structure. This was to be expected to be energy‐minimized. (Figure 6B).

**Figure 7:**
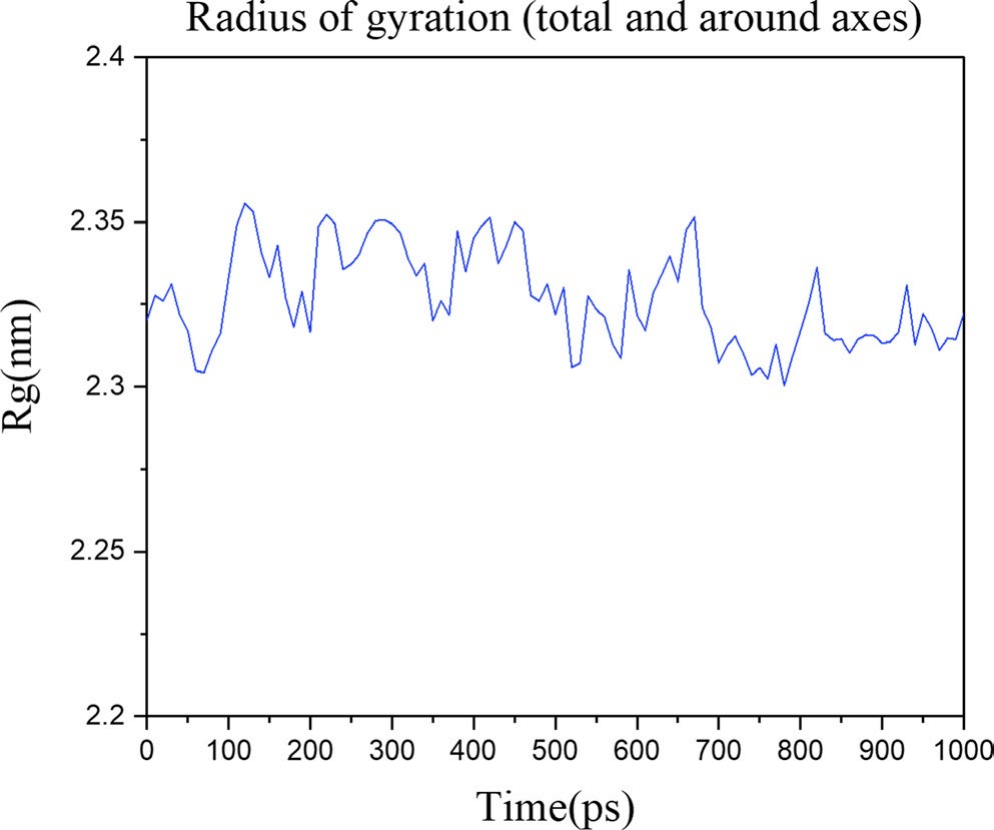
The radius of gyration of the ‘HLA‐A*0201~peptide’ complex

The radius of gyration (*Rg*) is a measure of its compactness. If a protein is stably folded, it will likely maintain a relatively steady value of *Rg*. We can see that over the course of 1 ns, the *Rg* value remains very stable, about 2.33 nm (Figure 7).

**Figure 8:**
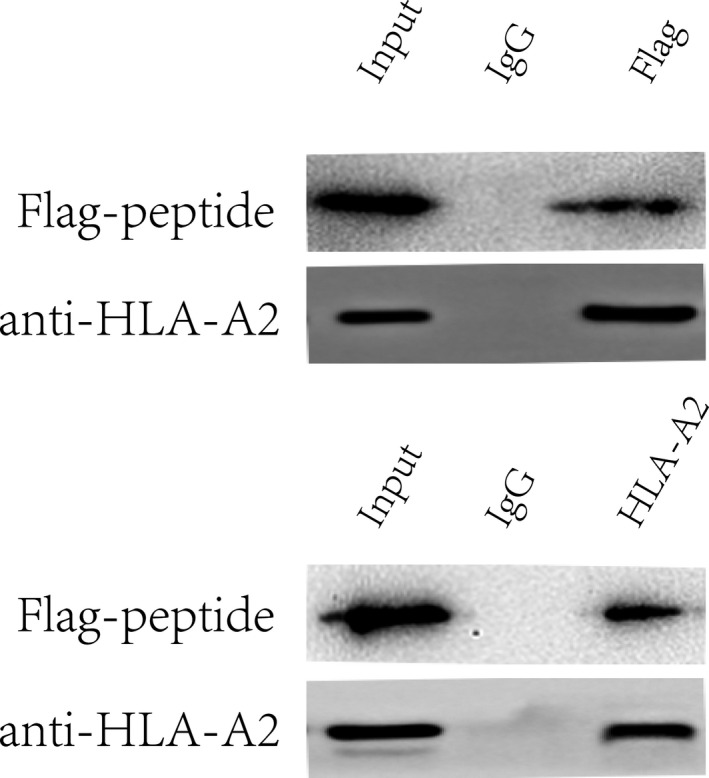
Co‐‐IP analysis of HLA‐A2 interacting with the peptide

Co‐IP analysis with anti‐Flag or anti‐HLA‐A2 antibody in T cells. Whole cell lysates were immunoprecipitated with an anti‐Flag or anti‐HLA‐A2 antibody and blotted with an anti‐HLA‐A2 or anti‐Flag antibody, respectively (Figure 8).

### 3.9 CD8^+^ T cell responses

**Figure 9:**
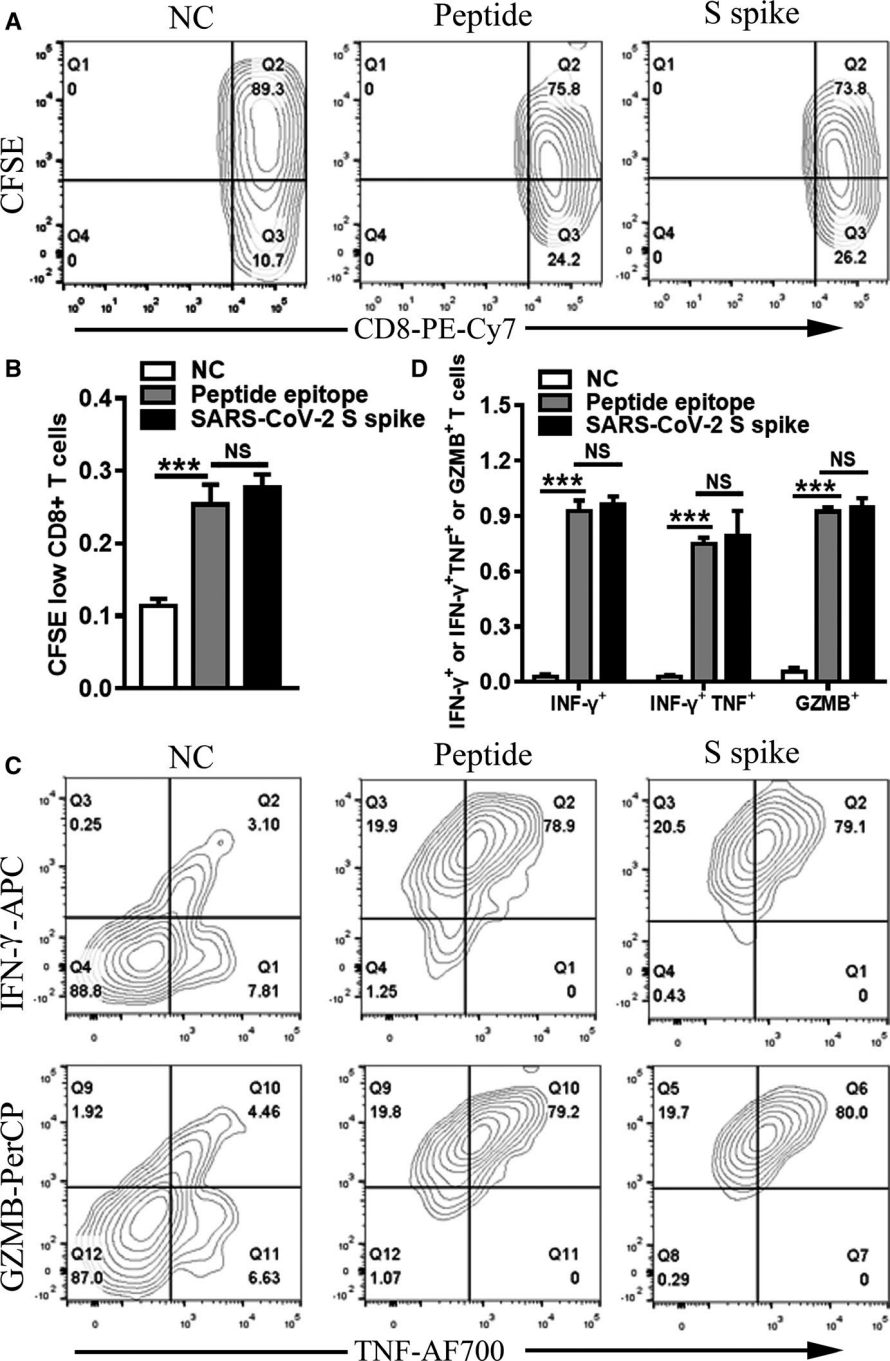
T cell proliferation and T cell response to the predicted peptide. Flow cytometry analysis(A) and statistics(B) of the percentage of CFSE low cells. Flow cytometric plots(C) representing CD8^+^ T cells expressing TNF‐α (x axis), IFN‐γ (y axis) and GZMB (y axis) upon stimulation with peptide and the SARS‐CoV‐2 S spike. And comparison(D) of TNF‐α, IFN‐γ and GZMB of the three groups (NC, Peptide and SARS‐CoV‐2 S spike). Student's t test was used for the analysis. ***P < 0.001

Proliferated T cells were identified by the percentage of CFSE low cells. The results showed that, compared with the negative control group, the predicted peptide could stimulate CD8^+^ T cell proliferation as significantly as S protein of SARS‐CoV‐2 (Figure 9A,B *P* < 0.001).

After identification of T cell proliferation to the peptide epitope, we further examined the specific CD8^+^ T cell response to the peptide. Cytokine‐producing T cells were detected by ICS after incubation with peptide or S protein of SARS‐CoV‐2. The responses detected against peptide were characterized by flow cytometry for peptide recognition by CD8^+^ T cell subsets and for intracellular production of IFN‐γ, tumour necrosis factor‐α (TNF‐α) and Granzyme B (GZMB) after stimulation. The results showed that the predicted peptide epitopes stimulated the expression of IFN‐γ, TNF‐α and GZMB significantly compared with the negative control group (Figure 9C,D *P* < 0.001).

## 4 DISCUSSION

SARS‐CoV‐2 has spread rapidly throughout the world and become a major threat to human health worldwide due to its extremely high transmissibility and pathogenicity. Therefore, there is an urgent need for effective therapy and prevention for COVID‐19. Its main clinical symptoms include varying degrees of pneumonia, impaired organ function and obvious gastrointestinal symptoms in some patients.^36^ The virus underwent rapid evolution after infection with host cells due to recombination between the genomes of different virus particles, which creates great difficulties for virus‐therapy, especially vaccine development. Vaccine development is also ongoing, without vaccines or drugs approved to treat COVID‐19, and many therapies with initial good clinical response are still being tested in clinical trials. In consequence, we hope to find a vaccine against SARS‐CoV‐2 that will lead to effective treatment and prevention strategies.

The S protein of coronavirus is vital for vaccine production. On the one hand, due to be exposed to the cell surface, it has an advantage antigenicity; on the other hand, S protein initiates viral infection by binding to the receptor ACE2.^37^ The S protein contains two subunits (S1 and S2). The S1 subunit can be further defined by two domains called N‐terminal domains (NTD) and C‐terminal domains (CTD), and the receptor‐binding domain (RBD) is in the CTD. Previous studies have shown that a large number of antibodies targeted RBD of SARS‐COV or MERS‐COV. But new research showed that the monoclonal antibody (mAb) isolated and identified from ten convalescent COVID‐19 patients didn't bind to RBD but to the N‐terminal domain (NTD) of S protein.^38^ These findings are particularly important for the development of a safe and effective COVID‐19 vaccine. The selection of vaccine binding targets also requires further research and screening.

However, one thing is certain: the viral S protein is the major target for neutralizing antibodies, the key point for the development of an effective vaccine and the focus of immunotherapy strategies for coronavirus. In our study, we compared viral S protein sequences from eight countries and found that they were highly conserved and rarely mutated. Then, we predicted the B cell and T cell epitopes of S protein through immunoinformatics and selected 10 B cell epitopes and 20 T cell epitopes (MHC‐1 and MHC‐II) according to the antigenicity of peptide epitopes. The peptide epitopes ‘EILDITPCSFGGVS’ at 583 showed the highest antigenicity (1.6193); ‘KIADYNYKL’ and ‘LEILDITPC’ had the highest antigenicity in T cell epitopes, which were, respectively, 1.6639 and 1.6390. At the same time, all of them showed a conversation of 100%, which indicates these peptides meet the prerequisites for peptide vaccines.

It should be noted that the binding of T cell receptor (TCR) and peptide‐Major histocompatibility complex (pMHC) is necessary for adaptive immunity, but this is driven by a number of factors. In fact, the signal strength of TCR was mainly limited by the binding topology of the ‘TCR‐peptide‐MHC’ complex.^39^ To be exact, the conformation of TCR and the recognition of MHC by TCR^40^ and different binding modes^41^ will affect T cell signalling, and it is not just the highly avid peptide antigen. Meanwhile, ‘TCR‐pMHC’ bond conformation controls TCR ligand discrimination.^42^ And structural interplay between germline interactions and adaptive recognition can also affect T cell receptor signalling.^43^ However, there is still a universality in ‘TCR‐pMHC’ recognition that TCR must recognize both MHC molecules and peptides.^44^

Meanwhile, SARS‐CoV‐2 infection can trigger strong T cell immunity in patients,^45^ and the first autopsy of SARS‐CoV‐2 patients showed the accumulation of lymphocytes in the lung.^46^ With SARS‐CoV‐2 infection, there are increasing evidences that virus‐specific T cells are indeed produced. It is worth noting that human leucocyte antigen (HLA) plays an important role in the immune response to viral infection. Moreover, studies have shown that HLA is associated with increased disease severity of SARS‐CoV‐2 infection.^47^, ^48^ Therefore, some researchers analysed the binding affinity between HLA and SARS‐CoV‐2 peptides through computer prediction, and then screened out many advantageous HLA.^49^ Further experiments have found that HLA Class I genotypes play an important role in SARS‐CoV‐2 infection and progression. Interestingly, HLA‐A2 supertypes recognize more peptides than A1 and A3 supertypes.^50^ The latest results showed that A2 /CD8 ^+^ T cells in SARS‐CoV‐2‐infected patients are activated and significantly amplified compared to healthy individuals. In addition, CD8^+^ T cells in convalescent HLA‐A *02:01 COVID‐19 patients also increased significantly in comparison with the controls.^51^

To further test the efficacy of our predicted peptide epitopes, we analysed all T‐ cell peptide epitopes for characteristics such as digestion, mutation, toxicity, sensitization, moisture and physicochemical properties. The comprehensive results showed that ‘KIADYNYKL’ is an ideal target for peptide vaccine because it not only has high antigenicity, but a possible surface antigen without toxicity and mutagenicity. The peptide vaccine is a combination of T cell and B cell epitopes, which can induce cell‐mediated and humoral immunity. And our predicted MHC class I peptide epitope ‘KIADYNYKL’ also overlaps with the predicted B cell epitope ‘GKIADYNYKLPDDF’, which makes it possible to participate in the immune activation of B cells and T cells at the same time. This is also a great result. In view of this, we chose it to carry on molecular docking with different MHC class I molecules. Meanwhile, T cells (helper T cells and cytotoxic T cells) are important mediators of cellular immunity. Antigen peptide‐MHC‐I complex can induce T cell activation, thereby activating the cell‐mediated immune response.^52^ Therefore, we performed the ‘MHC‐I molecule‐peptide epitope‐T cell TCR receptor’ multimeric complex simulation. Our results indicate that after the peptide ‘KIADYNYKL’ is bound to the MHC‐I molecules, it is possible to be presented to the T cell receptor and activate cellular immunity.

The peptide we selected ‘KIADYNYKL’ had a high affinity binding to HLA‐a*0201. However, this study has not yet conducted a combined response analysis of hybrid peptides among different HLA‐specific groups. For example, we predict that both MHC‐I and MHC‐II binding peptide ‘VVVLSFELL’ with HLA have a very good binding force. So the mixed peptide vaccine may be more effective, which deserves further study.

In conclusion, our results indicated that the peptide epitopes we predicted had high antigenicity, stability and conservatism and were potential targets for peptide vaccines. It is hoped that our results will help with ongoing vaccine development and in particular reduce the difficulties in vaccine design.

## CONFLICT OF INTEREST

The authors have declared no conflicts of interest.

## AUTHOR CONTRIBUTIONS

**Chen Ziwei:** Conceptualization (equal); Data curation (equal); Formal analysis (equal); Software (equal); Visualization (equal); Writing‐original draft (equal); Writing‐review & editing (equal). **Ruan Pinglang:** Conceptualization (equal); Data curation (equal); Formal analysis (equal); Software (equal); Visualization (equal); Writing‐original draft (equal); Writing‐review & editing (equal). **Wang Lili:** Supervision (supporting); Validation (supporting); Visualization (supporting); Writing‐original draft (supporting). **Nie Xinmin:** Supervision (supporting); Validation (supporting); Visualization (supporting); Writing‐original draft (supporting). **Ma Xuejun:** Conceptualization (equal); Data curation (equal); Formal analysis (equal); Funding acquisition (supporting); Resources (equal); Supervision (equal). **Tan Yurong:** Data curation (equal); Formal analysis (equal); Investigation (lead); Methodology (lead); Project administration (lead); Resources (equal); Supervision (equal); Validation (lead); Visualization (equal); Writing‐original draft (equal); Writing‐review & editing (lead).

## Supporting information

Fig S1‐5

Table S1‐5

## Data Availability

The data sets used and/or analysed during the current study are available from the corresponding author on reasonable request.

## References

[1] Chen N , Zhou M , Dong X , et al. Epidemiological and clinical characteristics of 99 cases of 2019 novel coronavirus pneumonia in Wuhan, China: a descriptive study. Lancet. 2020;395(10223):507‐513. 10.1016/S0140-6736(20)30211-7 32007143 PMC7135076

[2] Wu F , Zhao S , Yu B , et al. A new coronavirus associated with human respiratory disease in China. Nature. 2020;579(7798):265‐269. 10.1038/s41586-020-2008-3 32015508 PMC7094943

[3] Carlos WG , Dela Cruz CS , Cao B , Pasnick S , Jamil S . Novel Wuhan (2019‐nCoV) coronavirus. Am J Respir Crit Care Med. 2020;201(4):P7‐P8. 10.1164/rccm.2014P7 32004066

[4] Li Q , Guan X , Wu P , et al. Early transmission dynamics in Wuhan, China, of novel coronavirus‐infected pneumonia. N Engl J Med. 2020;382(13):1199‐1207. 10.1056/NEJMoa2001316 31995857 PMC7121484

[5] Ren JG , Li DY , Wang CF , et al. Positive RT‐PCR in urine from an asymptomatic patient with novel coronavirus 2019 infection: a case report. Infect Dis (Lond). 2020;52(8):571‐574. 10.1080/23744235.2020.1766105 32420777

[6] Wang FS , Zhang C . What to do next to control the 2019‐nCoV epidemic? Lancet. 2020;395(10222):391‐393. 10.1016/S0140-6736(20)30300-7 32035533 PMC7138017

[7] Wu Z , McGoogan JM . Characteristics of and important lessons from the Coronavirus Disease 2019 (COVID‐19) outbreak in China: summary of a report of 72314 cases from the Chinese Center for Disease Control and Prevention. JAMA. 2020 323(13):1239‐1242. 10.1001/jama.2020.2648 32091533

[8] Chan JF , Yuan S , Kok KH , et al. A familial cluster of pneumonia associated with the 2019 novel coronavirus indicating person‐to‐person transmission: a study of a family cluster. Lancet. 2020;395(10223):514‐523. 10.1016/S0140-6736(20)30154-9 31986261 PMC7159286

[9] Jiang S , Du L , Shi Z . An emerging coronavirus causing pneumonia outbreak in Wuhan, China: calling for developing therapeutic and prophylactic strategies. Emerg Microbes Infect. 2020;9(1):275‐277. 10.1080/22221751.2020.1723441 32005086 PMC7033706

[10] Velavan TP , Meyer CG . The COVID‐19 epidemic. Trop Med Int Health. 2020;25(3):278‐280. 10.1111/tmi.13383 32052514 PMC7169770

[11] Zhou P , Yang XL , Wang XG , et al. A pneumonia outbreak associated with a new coronavirus of probable bat origin. Nature. 2020;579(7798):270‐273. 10.1038/s41586-020-2012-7 32015507 PMC7095418

[12] de Wit E , van Doremalen N , Falzarano D , Munster VJ . SARS and MERS: recent insights into emerging coronaviruses. Nat Rev Microbiol. 2016;14(8):523‐534. 10.1038/nrmicro.2016.81 27344959 PMC7097822

[13] Le TT , Andreadakis Z , Kumar A , et al. The COVID‐19 vaccine development landscape. Nat Rev Drug Discov. 2020;19(5):305‐306. 10.1038/d41573-020-00073-5 32273591

[14] Folaranmi T . Use of Serogroup B meningococcal vaccines in persons aged >= 10 years at increased risk for Serogroup B meningococcal disease: recommendations of the advisory committee on immunization practices, 2015 (vol 64, pg 608, 2015). MMWR Morb Mortal Wkly Rep. 2015;64(29):806. PMC4584923 26068564

[15] Ul Qamar MT , Saleem S , Ashfaq UA , Bari A , Anwar F , Alqahtani S . Epitope‐based peptide vaccine design and target site depiction against Middle East Respiratory Syndrome Coronavirus: an immune‐informatics study. J Transl Med. 2019;17(1):362 10.1186/s12967-019-2116-8 31703698 PMC6839065

[16] Haveri A , Smura T , Kuivanen S , et al. Serological and molecular findings during SARS‐CoV‐2 infection: the first case study in Finland, January to February 2020. Euro Surveill. 2020;25(11):2000266 10.2807/1560-7917.ES.2020.25.11.2000266 PMC7096774 32209163

[17] Walls AC , Park YJ , Tortorici MA , Wall A , McGuire AT , Structure VD . Function, and antigenicity of the SARS‐CoV‐2 spike glycoprotein. Cell. 2020;181(2):281‐292.e6. 10.1016/j.cell.2020.02.058. 32155444 PMC7102599

[18] Gasteiger E , Gattiker A , Hoogland C , Ivanyi I , Appel RD , Bairoch A . ExPASy: The proteomics server for in‐depth protein knowledge and analysis. Nucleic Acids Res. 2003;31(13):3784‐3788. 10.1093/nar/gkg563 12824418 PMC168970

[19] Buchan DW , Minneci F , Nugent TC , Bryson K , Jones DT . Scalable web services for the PSIPRED Protein Analysis Workbench. Nucleic Acids Res. 2013;41:W349‐W357. 10.1093/nar/gkt381 23748958 PMC3692098

[20] Ferre F , Clote P . DiANNA 1.1: an extension of the DiANNA web server for ternary cysteine classification. Nucleic Acids Res. 2006;34:W182‐W185. 10.1093/nar/gkl189 16844987 PMC1538812

[21] Doytchinova IA , Flower DR . VaxiJen: a server for prediction of protective antigens, tumour antigens and subunit vaccines. BMC Bioinformatics. 2007;8:4 10.1186/1471-2105-8-4 17207271 PMC1780059

[22] Peters B , Sidney J , Bourne P , et al. The immune epitope database and analysis resource: from vision to blueprint. PLoS Biol. 2005;3(3):e91 10.1371/journal.pbio.0030091 15760272 PMC1065705

[23] Jespersen MC , Peters B , Nielsen M , Marcatili P . BepiPred‐2.0: improving sequence‐based B‐cell epitope prediction using conformational epitopes. Nucleic Acids Res. 2017;45(W1):W24‐W29. 10.1093/nar/gkx346 28472356 PMC5570230

[24] Koehl P , Levitt M . Structure‐based conformational preferences of amino acids. Proc Natl Acad Sci U S A. 1999;96(22):12524‐12529. 10.1073/pnas.96.22.12524 10535955 PMC22969

[25] Yao B , Zheng D , Liang S , Zhang C . Conformational B‐cell epitope prediction on antigen protein structures: a review of current algorithms and comparison with common binding site prediction methods. PLoS One. 2013;8(4):e62249 10.1371/journal.pone.0062249 23620816 PMC3631208

[26] Sun P , Ju H , Liu Z , et al. Bioinformatics resources and tools for conformational B‐cell epitope prediction. Comput Math Methods Med. 2013;2013:943636 10.1155/2013/943636 23970944 PMC3736542

[27] Janson G , Zhang C , Prado MG , Paiardini A . PyMod 2.0: improvements in protein sequence‐structure analysis and homology modeling within PyMOL. Bioinformatics. 2017;33(3):444‐446. 10.1093/bioinformatics/btw638 28158668

[28] Yang X , Yu X . An introduction to epitope prediction methods and software. Rev Med Virol. 2009;19(2):77‐96. 10.1002/rmv.602 19101924

[29] Singh H , Raghava GP . ProPred: prediction of HLA‐DR binding sites. Bioinformatics. 2001;17(12):1236‐1237. 10.1093/bioinformatics/17.12.1236 11751237

[30] Dimitrov I , Naneva L , Doytchinova I , Bangov I . AllergenFP: allergenicity prediction by descriptor fingerprints. Bioinformatics. 2014;30(6):846‐851. 10.1093/bioinformatics/btt619 24167156

[31] Sayers EW , Cavanaugh M , Clark K , Ostell J , Pruitt KD , Karsch‐Mizrachi I . GenBank. Nucleic Acids Res. 2020;48(D1):D84‐D86. 10.1093/nar/gkz956 31665464 PMC7145611

[32] Maupetit J , Derreumaux P , Tuffery P . PEP‐FOLD: an online resource for de novo peptide structure prediction. Nucleic Acids Res. 2009;37:W498‐W503. 10.1093/nar/gkp323 19433514 PMC2703897

[33] Dev S , Dhaneshwar SR , Mathew B . Discovery of camptothecin based Topoisomerase I Inhibitors: identification using an atom based 3D‐QSAR, pharmacophore modeling, virtual screening and molecular docking approach. Comb Chem High Throughput Screen. 2016;19(9):752‐763. 10.2174/1386207319666160810154346 27515040

[34] Pettersen EF , Goddard TD , Huang CC , et al. UCSF Chimera–a visualization system for exploratory research and analysis. J Comput Chem. 2004;25(13):1605‐1612. 10.1002/jcc.20084 15264254

[35] Garboczi DN , Ghosh P , Utz U , Fan QR , Biddison WE , Wiley DC . Structure of the complex between human T‐cell receptor, viral peptide and HLA‐A2. J Immunol. 2010;185(11):134‐141. 21084668

[36] Zhang JJ , Dong X , Cao YY , et al. Clinical characteristics of 140 patients infected with SARS‐CoV‐2 in Wuhan, China. Allergy. 2020;75(7):1730‐1741. 10.1111/all.14238 32077115

[37] Zhang J , Zeng H , Gu J , Li H , Zheng L , Zou Q . Progress and prospects on vaccine development against SARS‐CoV‐2. Vaccines (Basel). 2020;8(2):153 10.3390/vaccines8020153 PMC7349596 32235387

[38] Chi X , Yan R , Zhang J , et al. A neutralizing human antibody binds to the N‐terminal domain of the Spike protein of SARS‐CoV‐2. Science. 2020;369(6504):650‐655. 10.1126/science.abc6952 32571838 PMC7319273

[39] Adams JJ , Narayanan S , Liu BY , et al. T Cell receptor signaling is limited by docking geometry to peptide‐major histocompatibility complex. Immunity. 2011;35(5):681‐693. 10.1016/j.immuni.2011.09.013 22101157 PMC3253265

[40] Kalergis AM , Nathenson SG . Altered peptide ligand‐mediated TCR antagonism can be modulated by a change in a single amino acid residue within the CDR3 beta of an MHC class I‐Restricted TCR. J Immunol. 2000;165(1):280‐285. 10.4049/jimmunol.165.1.280 10861062

[41] Coles CH , Mulvaney RM , Malla S , et al. TCRs with distinct specificity profiles use different binding modes to engage an identical peptide‐HLA complex. J Immunol. 2020;204(7):1943‐1953. 10.4049/jimmunol.1900915 32102902 PMC7086387

[42] Sasmal DK , Feng W , Roy S , et al. TCR‐pMHC bond conformation controls TCR ligand discrimination. Cell Mol Immunol. 2020;17(3):203‐217. 10.1038/s41423-019-0273-6 31530899 PMC7052167

[43] Adams JJ , Narayanan S , Birnbaum ME , et al. Structural interplay between germline interactions and adaptive recognition determines the bandwidth of TCR‐peptide‐MHC cross‐reactivity. Nat Immunol. 2016;17(1):87‐94. 10.1038/ni.3310 26523866 PMC4684756

[44] La Gruta NL , Gras S , Daley SR , Thomas PG , Rossjohn J . Understanding the drivers of MHC restriction of T cell receptors. Nat Rev Immunol. 2018;18(7):467‐478. 10.1038/s41577-018-0007-5 29636542

[45] Tay MZ , Poh CM , Renia L , MacAry PA , Ng LFP . The trinity of COVID‐19: immunity, inflammation and intervention. Nat Rev Immunol. 2020;20(6):363‐374. 10.1038/s41577-020-0311-8 32346093 PMC7187672

[46] Xu Z , Shi L , Wang Y , et al. Pathological findings of COVID‐19 associated with acute respiratory distress syndrome. Lancet Respir Med. 2020;8(4):420‐422. 10.1016/S2213-2600(20)30076-X 32085846 PMC7164771

[47] Sanchez‐Mazas A . HLA studies in the context of coronavirus outbreaks. Swiss Med Wkly. 2020;150:w20248 10.4414/smw.2020.20248 32297958

[48] Olwenyi OA , Dyavar SR , Acharya A , et al. Immuno‐epidemiology and pathophysiology of coronavirus disease 2019 (COVID‐19). J Mol Med (Berl). 2020;98(10):1369‐1383. 10.1007/s00109-020-01961-4 32808094 PMC7431311

[49] Nguyen A , David JK , Maden SK , et al. Human leukocyte antigen susceptibility map for Severe Acute Respiratory Syndrome Coronavirus 2. J Virol. 2020;94(13):e00510‐20 10.1128/JVI.00510-20 32303592 PMC7307149

[50] Iturrieta‐Zuazo I , Rita CG , Garcia‐Soidan A , et al. Possible role of HLA class‐I genotype in SARS‐CoV‐2 infection and progression: A pilot study in a cohort of Covid‐19 Spanish patients. Clin Immunol. 2020;219:108572 10.1016/j.clim.2020.108572 32810602 PMC7428760

[51] Habel JR , Nguyen THO , van de Sandt CE , et al. Suboptimal SARS‐CoV‐2‐specific CD8(+) T cell response associated with the prominent HLA‐A*02:01 phenotype. Proc Natl Acad Sci U S A. 2020;117(39):24384‐24391. 10.1073/pnas.2015486117 32913053 PMC7533701

[52] Liu IH , Lo YS , Yang JM . PAComplex: a web server to infer peptide antigen families and binding models from TCR‐pMHC complexes. Nucleic Acids Res. 2011;39:W254‐W260. 10.1093/nar/gkr434 21666259 PMC3125798

