# Supplementary material for: T and B cell Epitope analysis of SARS‐CoV‐2 S protein based on immunoinformatics and experimental research
Source: J Cell Mol Med. 2020 Dec 15;25(2):1274–89. doi: 10.1111/jcmm.16200 (PMC7812294; doi:10.1111/jcmm.16200)

Supplementary pictures:

Figure S1. The secondary structure of SARS-COV-2 S protein.


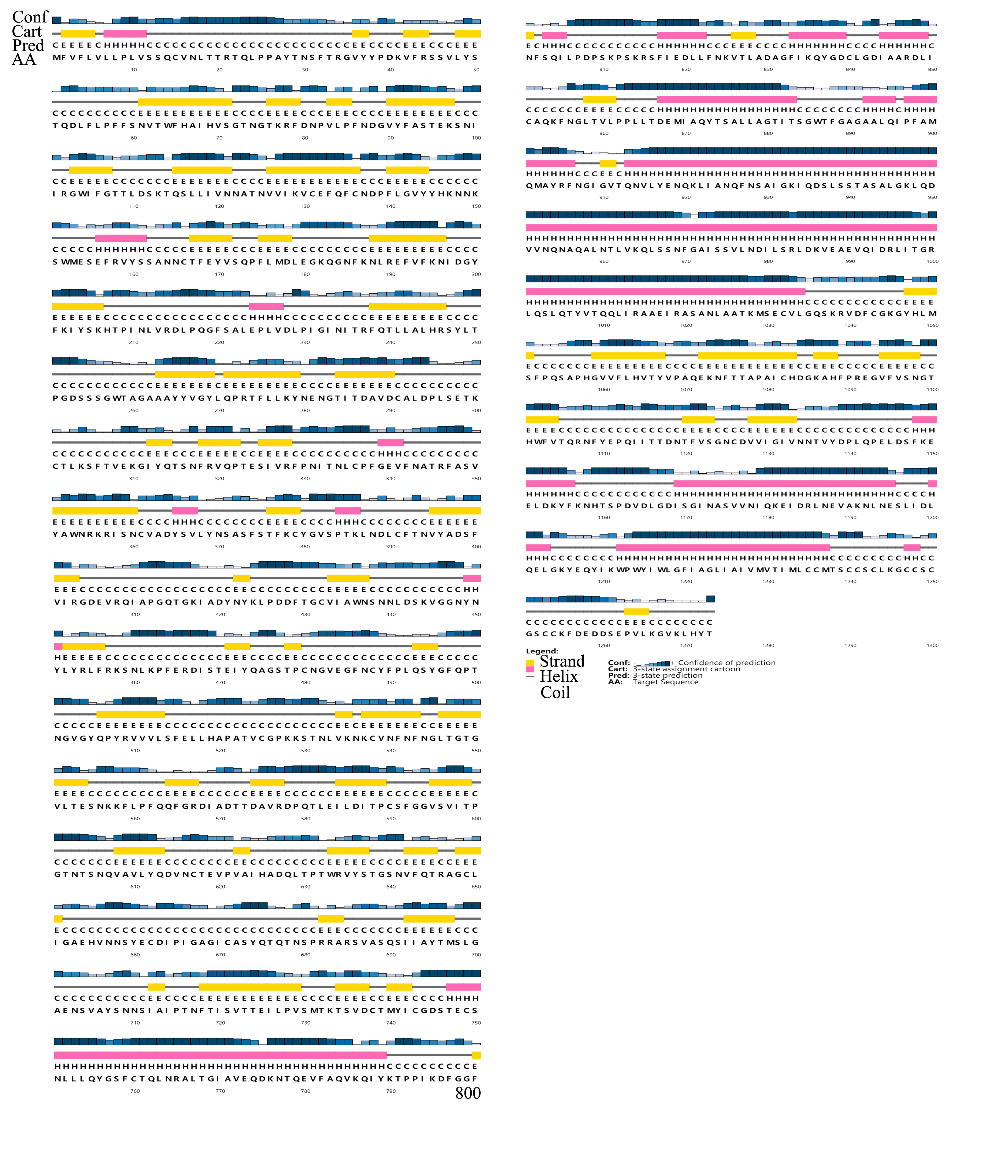


Figure S2. Sequence diagram of the secondary structure of SARS-COV-2 S protein.


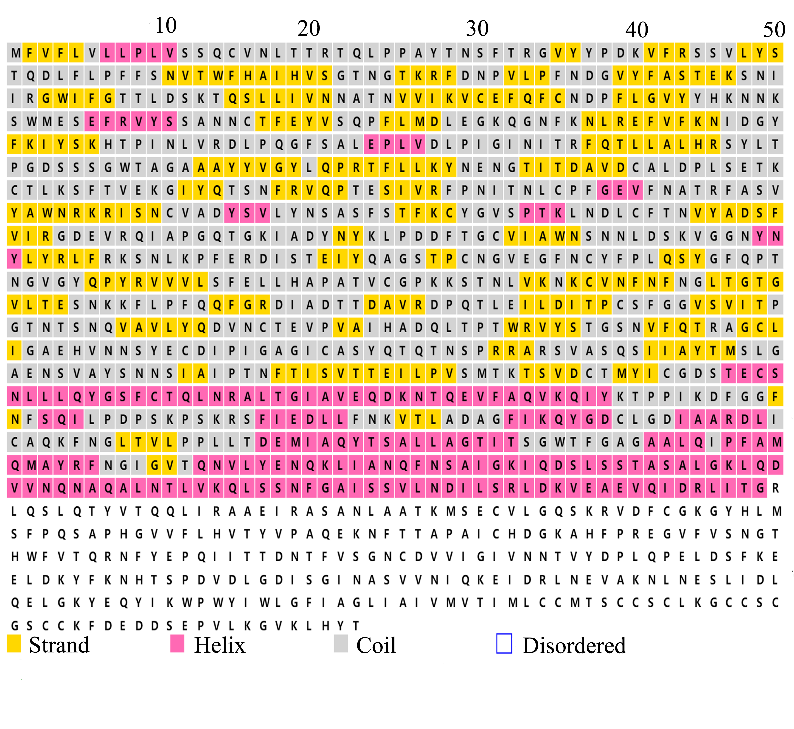


Figure S3. The 3D structure of the SARS-COV-2 S protein.


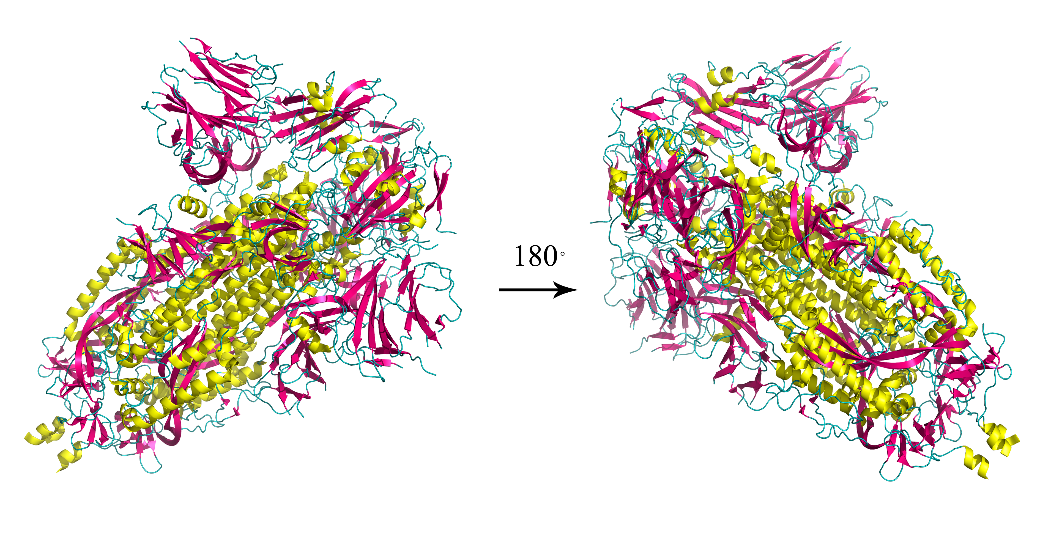


Figure S4. Comparison of SARS-CoV-2 S protein sequences from 8 different countries.


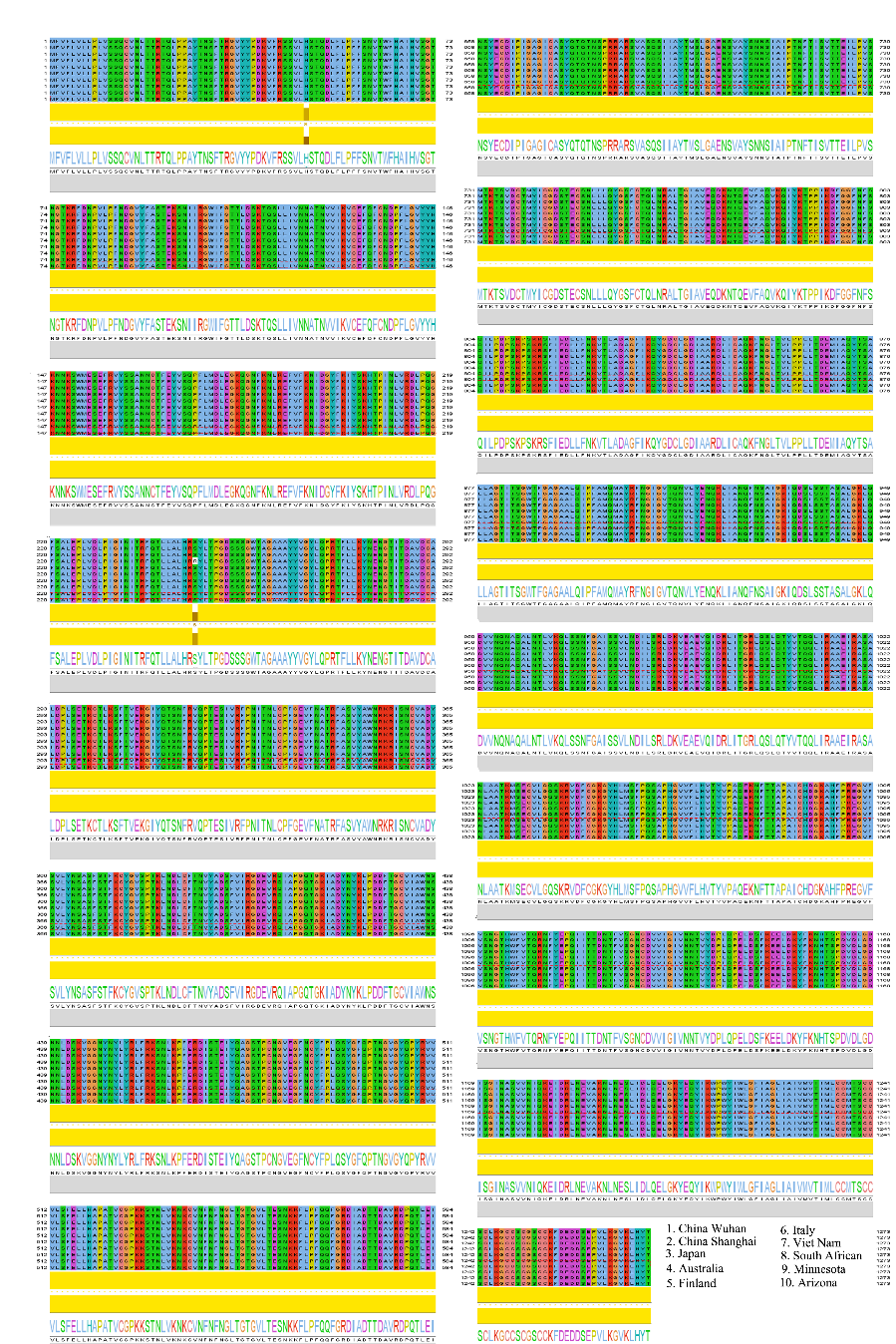


Figure S5. 3D model of MHC I peptide epitopes "KIADYNYKL"(A), "VVVLSFELL"(B) and “TLDSKTQSL”(C). 3D model (A1-3) and stick structures (B1-3).


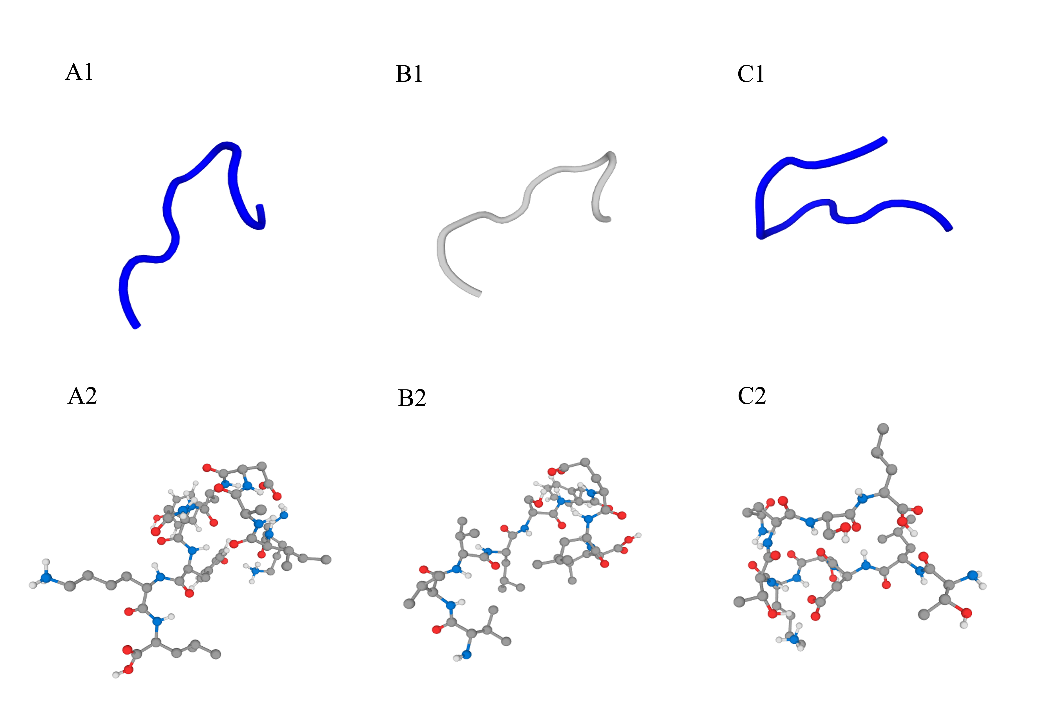

Supplement: Supplementary file 1 — Fig S1‐5 [file JCMM-25-1274-s001.docx]
