# Supplementary material for: T and B cell Epitope analysis of SARS‐CoV‐2 S protein based on immunoinformatics and experimental research
Source: J Cell Mol Med. 2020 Dec 15;25(2):1274–89. doi: 10.1111/jcmm.16200 (PMC7812294; doi:10.1111/jcmm.16200)
Supplement: Supplementary file 2 — Table S1‐5 [file JCMM-25-1274-s002.docx]

**Table S1: Physico-Chemical parameters of spike (S) protein computed through ExPASy ProtParam server.**

| **Parameters** | **S protein** |
| --- | --- |
| Mol. Weight | 141204.51 Dalton |
| No. of amino acids | 1273 |
| Theoretical *pI* | 6.21 |
| Instability index (II) | 33.01 (stable) |
| No. of Negatively Charged Residues (Asp + Glu) | 110 |
| No. of Positively Charged Residues (Arg + Lys) | 103 |
| Aliphatic Index | 84.67 |
| Grand average of Hydropathicity (GRAVY) | -0.078 |
| Atomic Composition | \| Hydrogen \| 9772 \| \| --- \| --- \| \| Nitrogen \| 1654 \| \| Oxygen \| 1895 \| \| Sulfur \| 54 \| |
| Amino Acid Composition | \| Arg (R) \| 42 \| 3.30% \| \| --- \| --- \| --- \| \| Asn (N) \| 88 \| 6.90% \| \| Asp (D) \| 62 \| 4.90% \| \| Cys (C) \| 40 \| 3.10% \| \| Gln (Q) \| 62 \| 4.90% \| \| Glu (E) \| 48 \| 3.80% \| \| Gly (G) \| 82 \| 6.40% \| \| His (H) \| 16 \| 1.30% \| \| Ile (I) \| 76 \| 6.00% \| \| Leu (L) \| 108 \| 8.50% \| \| Lys (K) \| 61 \| 4.80% \| \| Met (M) \| 14 \| 1.10% \| \| Phe (F) \| 77 \| 6.00% \| \| Pro (P) \| 58 \| 4.60% \| \| Ser (S) \| 99 \| 7.80% \| \| Thr (T) \| 97 \| 7.60% \| \| Trp (W) \| 12 \| 0.90% \| \| Tyr (Y) \| 55 \| 4.30% \| \| Val (V) \| 97 \| 7.60% \| \| Pyl (O) \| 0 \| 0.00% \| \| Sec (U) \| 0 \| 0.00% \| |

**Table S2: Predicted disulphide bonds within residues of S protein via DiANNA 1.1 web Server. The bonds with lowest Score indicated as red colours are weak bonds.**

| Sequence positions | Bonds | Scores |
| --- | --- | --- |
| 15 - 1240 | LVSSQCVNLTT - CCMTSCCSCLK | 0.97251 |
| 131 - 391 | VVIKVCEFQFC - KLNDLCFTNVY | 0.98595 |
| 136 - 662 | CEFQFCNDPFL - NNSYECDIPIG | 0.99337 |
| 166 - 1236 | SSANNCTFEYV - TIMLCCMTSCC | 0.99831 |
| 291 - 671 | TDAVDCALDPL - IGAGICASYQT | 0.90936 |
| 301 - 336 | LSETKCTLKSF - NITNLCPFGEV | 0.99406 |
| 361 - 488 | KRISNCVADYS - VEGFNCYFPLQ | 0.99701 |
| 379 - 743 | FSTFKCYGVSP - CTMYICGDSTE | 0.99720 |
| 432 - 1235 | DDFTGCVIAWN - VTIMLCCMTSC | 0.99881 |
| 480 - 1248 | AGSTPCNGVEG - CLKGCCSCGSC | 0.99870 |
| 525 - 1247 | APATVCGPKKS - SCLKGCCSCGS | 0.51364 |
| 538 - 1043 | LVKNKCVNFNF - KRVDFCGKGYH | 0.99952 |
| 590 - 617 | LDITPCSFGGV - YQDVNCTEVPV | 1.00000 |
| 649 - 1241 | QTRAGCLIGAE - CMTSCCSCLKG | 0.99965 |
| 738 - 1243 | KTSVDCTMYIC - TSCCSCLKGCC | 0.01037 |
| 749 - 1126 | GDSTECSNLLL - FVSGNCDVVIG | 0.99137 |
| 760 - 1250 | QYGSFCTQLNR - KGCCSCGSCCK | 0.80631 |
| 840 - 1032 | KQYGDCLGDIA - TKMSECVLGQS | 0.97557 |
| 851 - 1254 | ARDLICAQKFN - SCGSCCKFDED | 0.99959 |
| 1082 - 1253 | TAPAICHDGKA - CSCGSCCKFDE | 0.05754 |

**Table S3:** **Emini surface accessibility prediction results computed through IEDB Analysis Resource.**

| Position | Residue | Start | End | Peptide | Score |
| --- | --- | --- | --- | --- | --- |
| 79 | F | 77 | 82 | KRFDNP | 3.664 |
| 96 | E | 94 | 99 | STEKSN | 3.755 |
| 113 | K | 111 | 116 | DSKTQS | 3.899 |
| 146 | H | 144 | 149 | YYHKNN | 4.494 |
| 147 | K | 145 | 150 | YHKNNK | 5.736 |
| 148 | N | 146 | 151 | HKNNKS | 4.906 |
| 149 | N | 147 | 152 | KNNKSW | 3.791 |
| 280 | N | 278 | 283 | KYNENG | 3.613 |
| 459 | S | 457 | 462 | RKSNLK | 3.621 |
| 464 | F | 462 | 467 | KPFERD | 3.945 |
| 529 | K | 527 | 532 | PKKSTN | 5.003 |
| 555 | S | 553 | 558 | TESNKK | 5.603 |
| 676 | T | 674 | 679 | YQTQTN | 4.094 |
| 677 | Q | 675 | 680 | QTQTNS | 3.502 |
| 679 | N | 677 | 682 | QTNSPR | 4.243 |
| 680 | S | 678 | 683 | TNSPRR | 4.799 |
| 682 | R | 680 | 685 | SPRRAR | 4.091 |
| 683 | R | 681 | 686 | PRRARS | 4.091 |
| 775 | D | 773 | 778 | EQDKNT | 6.047 |
| 776 | K | 774 | 779 | QDKNTQ | 6.047 |
| 777 | N | 775 | 780 | DKNTQE | 6.047 |
| 809 | P | 807 | 812 | PDPSKP | 4.304 |
| 810 | S | 808 | 813 | DPSKPS | 3.73 |
| 811 | K | 809 | 814 | PSKPSK | 4.467 |
| 812 | P | 810 | 815 | SKPSKR | 5.658 |
| 813 | S | 811 | 816 | KPSKRS | 5.658 |
| 1071 | Q | 1069 | 1074 | PAQEKN | 3.919 |
| 1151 | E | 1149 | 1154 | KEELDK | 4.297 |
| 1155 | Y | 1153 | 1158 | DKYFKN | 3.791 |
| 1182 | E | 1180 | 1185 | QKEIDR | 3.577 |
| 1206 | Y | 1204 | 1209 | GKYEQY | 3.791 |
| 1257 | D | 1255 | 1260 | KFDEDD | 3.633 |
| 1259 | D | 1257 | 1262 | DEDDSE | 4.869 |
| 1260 | D | 1258 | 1263 | EDDSEP | 4.508 |

**Table S4:DiscoTope 2.0 prediction for structure: 6vxx.pdb.**

| Sr# | Residue ID | Residue Name | Contact Number | Propensity score | DiscoTope score |
| --- | --- | --- | --- | --- | --- |
| 1 | 282 | ASN | 5 | -2.384 | -2.685 |
| 2 | 415 | THR | 0 | -3.676 | -3.253 |
| 3 | 417 | LYS | 12 | -0.812 | -2.099 |
| 4 | 420 | ASP | 5 | -2.985 | -3.217 |
| 5 | 421 | TYR | 15 | -1.71 | -3.238 |
| 6 | 439 | ASN | 15 | -1.428 | -2.989 |
| 7 | 440 | ASN | 5 | -1.223 | -1.657 |
| 8 | 443 | SER | 19 | 0.432 | -1.803 |
| 9 | 444 | LYS | 8 | 1.79 | 0.664 |
| 10 | 447 | GLY | 14 | 2.779 | 0.849 |
| 11 | 448 | ASN | 27 | 1.336 | -1.922 |
| 12 | 449 | TYR | 3 | 0.877 | 0.431 |
| 13 | 450 | ASN | 15 | -0.334 | -2.021 |
| 14 | 452 | LEU | 11 | -2.239 | -3.247 |
| 15 | 454 | ARG | 14 | -0.401 | -1.965 |
| 16 | 462 | LYS | 4 | -3.237 | -3.324 |
| 17 | 467 | ASP | 19 | -1.575 | -3.579 |
| 18 | 468 | ILE | 6 | -2.698 | -3.078 |
| 19 | 489 | TYR | 0 | -0.418 | -0.37 |
| 20 | 490 | PHE | 9 | -0.819 | -1.76 |
| 21 | 491 | PRO | 11 | 0.123 | -1.156 |
| 22 | 492 | LEU | 11 | 1.006 | -0.374 |
| 23 | 493 | GLN | 11 | 1.042 | -0.342 |
| 24 | 494 | SER | 14 | 0.753 | -0.943 |
| 25 | 495 | TYR | 31 | 1.013 | -2.668 |
| 26 | 496 | GLY | 2 | 2.019 | 1.557 |
| 27 | 497 | PHE | 27 | 0.724 | -2.465 |
| 28 | 498 | GLN | 4 | 3.075 | 2.262 |
| 29 | 499 | PRO | 4 | 2.854 | 2.066 |
| 30 | 500 | THR | 1 | 4.501 | 3.868 |
| 31 | 501 | ASN | 24 | 3.882 | 0.676 |
| 32 | 503 | VAL | 2 | 0.217 | -0.038 |
| 33 | 504 | GLY | 3 | -2.009 | -2.123 |
| 34 | 505 | TYR | 10 | 0.727 | -0.506 |
| 35 | 506 | GLN | 14 | -0.912 | -2.417 |
| 36 | 556 | ASN | 0 | -3.192 | -2.825 |
| 37 | 558 | LYS | 0 | -2.012 | -1.781 |
| 38 | 560 | LEU | 4 | -3.135 | -3.235 |
| 39 | 703 | ASN | 3 | -2.229 | -2.318 |
| 40 | 704 | SER | 3 | -1.384 | -1.57 |
| 41 | 705 | VAL | 9 | -2.804 | -3.517 |
| 42 | 707 | TYR | 3 | -3.656 | -3.581 |
| 43 | 793 | PRO | 0 | -1.559 | -1.38 |
| 44 | 794 | ILE | 4 | -2.173 | -2.383 |
| 45 | 809 | PRO | 5 | -1.511 | -1.912 |
| 46 | 810 | SER | 4 | 1.115 | 0.526 |
| 47 | 811 | LYS | 20 | -0.982 | -3.169 |
| 48 | 812 | PRO | 3 | 0.186 | -0.18 |
| 49 | 914 | ASN | 7 | -0.803 | -1.516 |
| 50 | 917 | TYR | 9 | -2.704 | -3.428 |
| 51 | 918 | GLU | 11 | -2.415 | -3.402 |
| 52 | 1071 | GLN | 4 | -3.256 | -3.342 |
| 53 | 1074 | ASN | 8 | -2.813 | -3.409 |
| 54 | 1100 | THR | 0 | -3.398 | -3.007 |
| 55 | 1114 | ILE | 7 | -3.262 | -3.692 |
| 56 | 1118 | ASP | 6 | -3.105 | -3.438 |
| 57 | 1140 | PRO | 8 | -0.752 | -1.586 |
| 58 | 1141 | LEU | 3 | -0.71 | -0.974 |
| 59 | 1142 | GLN | 7 | 0.439 | -0.416 |
| 60 | 1143 | PRO | 6 | 0.445 | -0.296 |
| 61 | 1144 | GLU | 4 | 0.586 | 0.059 |
| 62 | 1145 | LEU | 5 | -0.203 | -0.754 |
| 63 | 1146 | ASP | 6 | 1.013 | 0.206 |
| 64 | 1147 | SER | 5 | -0.017 | -0.59 |

|  | **Sequences** | **Epitope length** | **Identity(10/10)** | |
| --- | --- | --- | --- | --- |
| **B-Cells peptides** | | | | |
| 1 | GKIADYNYKLPDDF | 14 | | 100% |
| 2 | FSTFKCYGVSPTKL | 14 | | 100% |
| 3 | ILPVSMTKTSVDCT | 14 | | 100% |
| 4 | AGCLIGAEHVNNSY | 14 | | 100% |
| 5 | QIPFAMQMAYRFNG | 14 | | 100% |
| 6 | LSSTASALGKLQDV | 14 | | 100% |
| 7 | SNVFQTRAGCLIGA | 14 | | 100% |
| 8 | DLPIGINITRFQTL | 14 | | 100% |
| 9 | EILDITPCSFGGVS | 14 | | 100% |
| 10 | VNFNFNGLTGTGVL | 14 | | 100% |
| **MHC class-I binding peptides** | | | | |
| 1 | KIADYNYKL | 9 | | 100% |
| 2 | TNFTISVTT | 9 | | 100% |
| 3 | VVVLSFELL | 9 | | 100% |
| 4 | TLDSKTQSL | 9 | | 100% |
| 5 | GKQGNFKNL | 9 | | 100% |
| 6 | VRDLPQGFS | 9 | | 100% |
| 7 | PWYIWLGFI | 9 | | 100% |
| 8 | NFGAISSVL | 9 | | 100% |
| 9 | QGFSALEPL | 9 | | 100% |
| 10 | NHTSPDVDL | 9 | | 100% |
| **MHC class-II binding peptides** | | | | |
| 1 | LEILDITPC | 9 | | 100% |
| 2 | LPVSMTKTS | 9 | | 100% |
| 3 | VVFLHVTYV | 9 | | 100% |
| 4 | YYVGYLQPR | 9 | | 100% |
| 5 | VVLSFELLH | 9 | | 100% |
| 6 | VVIGIVNNT | 9 | | 100% |
| 7 | YVGYLQPRT | 9 | | 100% |
| 8 | VNLTTRTQL | 9 | | 100% |
| 9 | IGINITRFQ | 9 | | 100% |
| 10 | LVKNKCVNF | 9 | | 100% |

**Table S5: Conservancy results of B-cells and T-cells (MHC Class-I and II) epitopes among all SARS-COV-2 isolates of different countries have been shown. The analyses were done using the IEDB Analysis Resource.**
